# Performance and feasibility of reactive surveillance and response strategies for malaria elimination in Vietnam: a mixed-methods study

**DOI:** 10.1186/s12936-023-04660-w

**Published:** 2023-08-07

**Authors:** Win Han Oo, Xuan Thang Nguyen, Thi Van Anh Ngo, Duc Thang Ngo, Win Htike, Nilar Aye Tun, Kaung Myat Thu, Julia Cutts, T Hong Phuc Nguyen, May Chan Oo, Ei Phyu Htwe, Aung Khine Zaw, Katherine O’Flaherty, Paul A. Agius, Freya J. I. Fowkes

**Affiliations:** 1 Disease Elimination Programme, Burnet Institute, 85 Commercial Rd, Melbourne, VIC Australia; 2 Health Security Programme, Burnet Institute Myanmar, 226 U Wisara Road, Yangon, Myanmar; 3 Department of Epidemiology, National Institute of Malariology, Parasitology and Entomology, Hanoi, Vietnam; 4 Health Poverty Action, Hanoi, Vietnam; 5 Centre for Epidemiology and Biostatistics, Melbourne School of Population and Global Health, University of Melbourne, Melbourne, VIC Australia; 6 Department of Epidemiology and Preventive Medicine, Monash University, Melbourne, VIC Australia

**Keywords:** Case notification, Case investigation, Focus investigation, Malaria elimination, Reactive surveillance and response, Reactive case detection, Vietnam

## Abstract

**Background:**

To enhance malaria elimination, Vietnam adopted a Reactive Surveillance and Response (RASR) Strategy in which malaria case notification and investigation must be completed within 2 days followed by a focus investigation within 7 days. The nationwide performance of Vietnam’s RASR strategy has yet to be evaluated. This study aims to evaluate the performance and feasibility of RASR in Vietnam, thereby providing recommendations for improved RASR.

**Methods:**

To assess malaria RASR in Vietnam, a mixed-methods study of (1) secondary data analysis of nationwide malaria case-based dataset from 2017 to 2021; (2) a quantitative survey, and (3) qualitative in-depth interviews and focus group discussions administered to central, provincial and district level stakeholders/staff and to the commune and community level front line health services providers was conducted.

**Results:**

In Vietnam, there are guidelines and procedures for implementation of each step of RASR. The completeness of case notification on the reported monthly aggregated data was very high in both the paper-based (12,463/12,498, 99.7% in 2017–2020) and electronic reporting systems (467/467, 100% in 2021 when electronic reporting was introduced); however, there were delays in notification while using the paper-based system (timely notification—7,978/12,498, 63.8%). In 2021, the completeness (453/467, 97.0%) and timeliness (371/467, 79.4%) of case investigation were found to be high. Reactive case detection was the major focus investigation response, with fever screening achievement of 88.6% (11,481 / 12,965) and 88.5% (11,471 / 12,965) among index case and neighbouring household members, respectively.

**Conclusions:**

Overall, there was policy commitment for implementation of RASR in Vietnam. The completeness and timeliness of case notification and case investigation were high and improved after the introduction of the electronic reporting system. More evidence is required for reactive case detection in defining the screening area or population.

**Supplementary Information:**

The online version contains supplementary material available at 10.1186/s12936-023-04660-w.

## Background

Vietnam, like other Greater Mekong Subregion (GMS) countries, aims to eliminate malaria by 2030 [1]. Significant progress toward this goal has been made with substantial declines in the malaria burden in Vietnam over the past two decades [2]; in 2020, Vietnam performed 1,811,387 malaria tests reporting 1421 malaria cases out of which 303 were imported cases [3].

Once the number of malaria cases is reduced to low levels, the priorities and activities of malaria programmes may need to be adjusted in order to achieve elimination [4]. Surveillance should be enhanced to ensure that every infection is detected and reported in a timely manner, and strategies for targeting both parasites and vectors should be implemented effectively in order to interrupt local malaria transmission [4]. Therefore, it is pragmatic to establish a case-based surveillance and response system to ensure every malaria case is investigated in order to understand risk factors and eliminate foci of transmission [5]. In this system, interventions must become increasingly “granular” allowing the identification, tracking and classification of all malaria cases and implementation of appropriate response activities [4, 5]. These activities are interconnected and are referred to as case detection and notification, case investigation and classification, and focus investigation and response including reactive case detection (RACD), a form of active case detection that focuses on screening of people geographically or characteristically associated with the index case of malaria [6, 7]” or herein collectively referred to as “Reactive surveillance and Response (RASR)”.

The National Institute of Malariology, Parasitology and Entomology (NIMPE), Vietnam has developed a RASR strategy by adapting the World Health Organization manual on malaria surveillance, monitoring and evaluation [8] that reflects Vietnam’s context and is acceptable to Vietnam Ministry of Health. As per the “Decision 741” issued by the Vietnam Ministry of Health on 2 March 2016 [9], all malaria suspected cases must be confirmed with microscopy, and reported within 2 days from detection and investigated within 3 days, respectively. Although there is no specific deadline in terms of the number of days after detection of an index case to complete focus investigation, if local transmission is detected or suspected, it must be done immediately after case investigation. The focus investigation and response includes thorough investigation of the case-detected area for receptivity and vulnerability for malaria transmission including entomological investigations and surveillance, and for available health services followed by classification of focus (active, residual non-active or clear focus), and handing the focus as per the classified type of focus. RACD is also implemented as a part of focus response among 20–30 neighbour households surrounding the index household [9]. An updated decision, “Decision 4922”, has been published on 25 October 2021 [10] in which either Rapid Diagnostic Test (RDT), microscopy or polymerase chain reaction (PCR) is allowed for confirmation of a malaria case. Case notification and investigation must be completed within 2 days, followed by the aforementioned focus investigation and response within 7 days.

The nationwide performance of Vietnam’s RASR has yet to be evaluated. This study sought to evaluate the performance and feasibility of the implementation of all RASR activities in Vietnam since the introduction of Decision 741 in 2016 (case notification, investigation and classification, and focus investigation and response activities) in order to provide recommendations for improved RASR in terms of quality, effectiveness, and coverage in the context of existing national health system which will contribute to achieving malaria elimination goal in Vietnam and more broadly in the GMS.

## Methods

### Study design and setting

A mixed-methods study including secondary data analysis of a national malaria case-based dataset aggregated from case reports, quantitative survey, and qualitative in-depth interviews (IDI) and focus group discussions (FGD) was conducted to assess malaria RASR in Vietnam. The reporting of the study adhered to the Strengthening the Reporting of Observational studies in Epidemiology checklists [11] (Additional file 1).

### Secondary data analysis

Data analysis of malaria case notification, case investigation, focus investigation, reactive surveillance and response data from NIMPE was conducted. The dataset (Additional file 2) that contains data on malaria cases was extracted on 18 March 2022. Data reported from communes, districts and provinces to NIMPE between 1 January 2017 and 31 December 2020 was extracted from the Excel Database (reported via paper-based forms) and those between 1 January and 31 December 2021 was extracted from electronic Communicable Disease Surveillance System—Malaria Management System (eCDS-MMS) (reported using the application on the eCDS—MMS webpage). The datasets were then imported to Stata and managed and cleaned for data analysis in Stata.

### Survey and qualitative consultations

For primary quantitative and qualitative data collections, Phu Yen and Binh Thuan Provinces were chosen as these provinces have been implementing malaria elimination activities including RASR strategies with the funding support from an international funding body, presence of participants who could provide valuable information of RASR strategies and operational feasibility of the budget and logistic arrangement.

### Quantitative survey

A cross-sectional quantitative survey was conducted using Questionnaire 1 and 2 (Additional file 3) in Phu Yen and Binh Thuan Provinces with 74 people (36 health stakeholders/staff and 38 frontline health services providers (FHSPs)). The majority of health stakeholders/staff surveyed were male and malaria programme management staff while the majority of participants in the FHSP survey were female clinical service providers such as medical doctors, nurses, midwives, health centre staff and village health workers (VHWs) (Additional file 5: Table S1).

Two stage sampling was applied by selecting the health facilities that implement RASR since March 2016 as per Decision 741 in the endemic areas in the two provinces followed by purposive selection of participants who have experiences in malaria control and elimination in the selected health facilities. Given the purposive selection of all available participants in the selected health facilities and intended descriptive analysis of the survey data, no formal sample size calculation was done.

### In-depth interviews and focus group discussions

Qualitative data collection was conducted in health stakeholders/staff and key experts at national and provincial levels (Phu Yen and Binh Thuan Provinces), FHSPs at district (Dong Xuan and Bac Binh districts) and commune levels, and mobile and migrant population (MMPs) including forest goers, who often go to the field sites or forest or who travel to another country or province in the community. MMPs who had ever suffered from malaria at least one time were selected by commune health centre staff from villages in the two provinces that had malaria cases. A total of 70 people (34 FHSPs and 36 MMPs, all participants were gender balanced) participated in the FGDs with approximately 4 to 5 participants per FGD in both provinces (Additional file 5: Table S2). A FGD guide (Additional file 4) was used to explore FHSP and MMPs’ opinions regarding implementing case notification, investigation and focus response, and barriers and enablers in its implementation. Applying the phenomenological approach, 28 semi-structured IDI were conducted using the topic guide (Additional file 4) to explore national, provincial and district level stakeholders/staff’s experiences in management of RASR as well as in development of RASR strategies. Interviewees were purposively recruited by NIMPE researchers based on their roles managing malaria reporting data and to ensure representation from multiple organizational levels (Additional file 5: Table S2).

### Data collection

The quantitative and qualitative primary data collections were conducted between November 2021 and April 2022. Data collection was completed in Vietnamese language. Staff of NIMPE performed face to face interviews using the paper questionnaires for survey, and FGD and IDI topic guides for qualitative data collection. The survey, IDI and FGD data collections approximately lasted for 45, 60 and 90 min respectively. All data collection happened in private locations where privacy was maintained.

### Data management and analyses

#### Secondary data analysis

Outcomes of interest were completeness and timeliness in case notification and investigation as these reflect the effectiveness of surveillance system in the malaria elimination programme. Derivation of completeness indicator for notification was straightforward; based on whether there was a date of reporting. Regarding completeness of case investigation, eCDS—MMS database contains date of case investigation, but in Excel database, case classification variable is taken as a proxy for completeness of case investigation given a malaria case could only be classified after case investigation and classification of a case indicates that this case is being investigated. Timeliness in malaria notification and case investigation were derived from the dates of test result, date of reporting and date of case investigation. Descriptive analysis was done for reporters having a phone number or an email address, malaria parasite species notified, case investigation and case classification, focus investigation and RACD; these categorical variables are shown in frequency (%). The list of variables is not consistent between the two databases of 2017–20 (Excel database) and 2021 (eCDS—MMS database) and some malaria programme indicators such as timeliness of case investigation, GPS mapping, follow-up on adherence of anti-malarial drugs, types of malaria diagnostic methods used cannot be elucidated for all the 5-year period.

#### Quantitative survey

The survey data collected (Additional file 5: Table S1) were typed into the Excel data spreadsheet formatted as per the variables in the Questionnaire 1 and 2. The dataset was imported into Stata for data cleaning, management and descriptive analyses. Data were categorized and analysed as per the thematic areas of case notification, case investigation and classification, and focus investigation including RACD.

#### In-depth interviews and focus group discussions

The FGDs and IDIs (Additional file 5: Table S2) were audio recorded and transcribed verbatim, translated into English, organized, managed, and analysed thematically (deductive followed by inductive analysis) [12]. A deductive thematic framework that includes coding definitions, themes and subthemes were developed before the analysis. Two researchers immersed the data by reading the transcripts three times. An inter-coder reliability test was done before coding. Each of the two researchers then coded data separately referring the deductive thematic framework. After coding, the researchers then discussed themes and subthemes to reach a consensus on the final thematic framework and interpretation [13].

Quantitative and qualitative data analyses were conducted in Stata Version 16.1 (StataCorp, Texas, USA) and Nvivo version 12, respectively. The results were reported thematically and as per the outcomes of performance and feasibility of implementing RASR strategies in Vietnam.

## Results

### Overall reactive surveillance and response strategies implemented in Vietnam

Almost all survey participants (68/71, 91.9%) confirmed that there is a time bound RASR strategy, either 2-3-7 strategy (case notification within 2 days, case investigation within 3 days and focus investigation within 7 days) or an adapted 2-7 strategy (case notification and investigation within 2 days and focus investigation within 7 days), applied in all areas (41/71, 57.8%) or elimination areas (19/71, 26.8%) of Vietnam (Additional file 5: Table S3). According to FGDs and IDIs, case notification is primarily done by VHWs and commune level FHSPs, and case investigation for identification of whether locally acquired or imported case is executed by commune level FHSPs supervised by district level FHSPs. Focus investigations and responses that included RACD, entomological surveillance and vector control measures were led by district level FHSPs supervised by provincial level staff. The focus is then monitored by the province level staff after one month of index case detection and continued to be monitored weekly until there is no more local transmission in the focus.

Although there were established RASR strategies, some FHSPs in FGDs were not aware of the detailed instructions for RASR mentioned in the National Malaria Surveillance Guidelines. When they were asked, some could not correctly name the latest Decisions (Surveillance Guidelines), Decision 4922, and explain the detailed instructions in the Guidelines.

*“Facilitator: Do you know the newest guidelines on surveillance, prevention and control of malaria?*

*FHSP: The Decision 2699.*

*Facilitator: How about the surveillance and prevention?*

*FHSP: I don’t remember.”* (A conversation in FGD with FHSPs, Phu Yen Province).

*“There is a procedure (for RASR), but I haven’t studied and read it yet”* (VHW, Binh Thuan Province). They justified that they must implement many programmes in the field while each programme demands many data that caused burden for them. *“I don’t remember. Because there are many other disease control programs in our commune: tuberculosis, eye diseases, infectious diseases such as dengue fever, *etc*. I don’t have information about all documents. Different documents are given to our commune health centre every year because we go to the District Health Centre for training each year”* (Commune level FHSP, Phu Yen Province).

Nevertheless, many FHSPs in FGDs in both provinces were able to recall the time schedule of Decision 4922 correctly and had expressed intension to follow the instructions on case notification, case investigation and focus investigation and response.

### Case notification

Secondary data was obtained from malaria case-based data reported as per the Decision 741 [9] and Decision 4922 [10]. A total of 12,498 malaria cases were reported between January 2017 and December 2020, with 467 malaria cases reported in 2021 (Additional file 5: Table S4). According to secondary data analysis, high levels of completeness were achieved with both paper-based (12,463/12,498, 99.7%, 2017–2020) and electronic reporting systems (eCDS—MMS introduced in 2021, 467/467, 100%). However, only 63.8% (7,978/12,498) of cases reported with paper-based reporting were notified in a timely manner (i.e., within 48 h as per the Decision 4922), increasing to 83.7% (391/467) after the introduction of eCDS – MMS in 2021 (Table 1).

**Table 1:** Malaria case notification and investigation in Vietnam from 2017 to 2021 (Secondary data analysis)

	Paper-based system^a^	eCDS – MMS^b^	Total
	n = 12,498	n = 467	n = 12,965
Completeness in malaria notification			
No	35 (0.3%)	0 (0.0%)	35 (0.3%)
Yes	12,463 (99.7%)	467 (100.0%)	12,930 (99.7%)
Delay in notification			
No	7978 (63.8%)	391 (83.7%)	8369 (64.6%)
Yes (> 48 h)	4473 (35.8%)	0 (0.0%)	4473 (34.5%)
Unable to evaluate due to data error	3 (0.0%)	2 (0.4%)	5 (0.0%)
Missing^d^	44 (0.4%)	74 (15.9%)	118 (0.9%)
Completeness in malaria case investigation			
No	732 (5.9%)	14 (3.0%)	746 (5.8%)
Yes	11,766 (94.1%)	453 (97.0%)	12,219 (94.2%)
Delay in case investigation^c^			
No	-na-	371 (79.4%)	371 (2.9%)
Yes (> 48 or 72 h)^c^	-na-	20 (4.3%)	20 (0.2%)
Unable to evaluate due to data error	-na-	1 (0.2%)	1 (0.0%)
Missing	12,498 (100.0%)	75 (16.1%)	12,573 (97.0%)
Classification of malaria patients			
Indigenous malaria case	7715 (61.7%)	356 (76.2%)	8,071 (62.3%)
Commune imported	1209 (9.7%)	83 (17.8%)	1292 (10.0%)
District imported	425 (3.4%)	0 (0.0%)	425 (3.3%)
Provincial imported	1824 (14.6%)	0 (0.0%)	1824 (14.1%)
Overseas imported	593 (4.7%)	5 (1.1%)	598 (4.6%)
Secondary transmission	0 (0.0%)	4 (0.9%)	4 (0.0%)
Relapse	0 (0.0%)	7 (1.5%)	7 (0.1%)
Case classification was not performed	732 (5.9%)	12(2.6%)	744 (5.7%)
Origin of imported case			
Indigenous malaria case	7715 (61.7%)	356 (76.2%)	8071 (62.3%)
Imported within Vietnam	3457 (27.7%)	83 (17.8%)	3,541 (27.3%)
From GMS countries	486 (3.9%)	1 (0.2%)	487 (3.8%)
Other countries outside of GMS	107 (0.9%)	4 (0.9%)	111 (0.9%)
Not recorded	733 (5.9%)	23 (4.9%)	756 (5.8%)

^a^Microsoft Excel-based dataset for malaria case-based data collected via paper-based reporting system between January 2017 and December 2020

^b^Electronic Communicable Disease Surveillance (eCDS – MMS) system used in 2021

^c^Case investigation must be done within 72 h from test result until Oct 2021. From Nov 2021, it must be done within 48 h

^d^Either one of the dates (date of result of the malaria diagnosis or date of reporting of malaria case) was missing

In the surveys, more than half (42/74, 57.5%) of the malaria stakeholders/staff and FHSPs stated that more than 90% of malaria case were notified within 24 h (although the standard time of notification is 48 h) after diagnosis (Additional file 5: Table S5). This may be due to the encouragement and pressure from the higher-level health stakeholders/staff for as early as possible and timely notification. *“The case notification needs to be implemented within 48 h. After 48 h, higher level will not accept the notification, therefore, it must be done in time. The earlier, the better”* (FHSP, Binh Thuan Province). According to discussions in FGDs, it took approximately one hour to complete the notification. Normally, FHSPs fill in the notification form electronically at one time in a day and send it out the next day.

Enablers and barriers to timely malaria case notification were identified in the secondary data analysis, surveys and qualitative consultations. Secondary data analysis of the national malaria case database demonstrated that over the 5-year, almost all (n = 12,104 / 12,965, 93.4%) malaria cases reported had a corresponding phone number of the person notifying the case, and almost three quarters (n = 327/ 467, 70.0%) of reported malaria cases had a corresponding email address of the person notifying the case, acting as a proxy for data entry on the webpage of eCDS—MMS in 2021. According to the FHSP survey, the majority of respondents mentioned that their village has mobile phone network coverage (36/38, 94.7%) and internet access (30/38, 79.0%), and 83.3% (25/30) of participants confirmed that the quality of both mobile phone and internet connection was good. Nearly a third of FHSPs (11/38, 29.0%) received a phone call from suspected malaria patients about their illness (Table 2). Nevertheless, FHSPs could not notify malaria cases detected in the field sites and forests outside of the village in time due to limited network coverage outside the village. Hence, they could only report the malaria case when they arrived back to the village. *“The difficulty is that there is no mobile network when we are in the field. We can’t update (malaria cases) immediately (in the mobile phone application). We can only update (the application) when we get the internet access”* (VHW, Binh Thuan Province).

**Table 2:** Characteristics of village/worksite operated by FHSPs (Survey)

Characteristic	Number (N = 38)	Percent
Number of households, mean (SD)	667.4 (878.8)	-na-
Population size, mean (SD)	2208.6 (2445.2)	-na-
Presence of mobile phone signal		
Yes	36	94.7
No	2	5.3
Quality of mobile phone signal		
Good	30	83.3
Poor	6	16.7
Presence of internet access		
Yes	30	79.0
No	8	21.1
Quality of internet access		
Good	25	83.3
Poor	5	16.7
Notification method for possible cases of malaria to the service provider		
Phone call from patient	11	29.0
Phone call/referral from patient’s family member or friend	1	2.6
Patient’s visit to volunteer	16	42.1
House visit by volunteer	12	31.6
Mass testing	6	15.8

Similarly, health stakeholders/staff and FHSPs mentioned in the survey that malaria cases were first reported to their supervisor or respective organization via phone call (27/74, 38.6%) or through an electronic reporting system (34/74, 48.6%). According to FGDs, FHSPs at communes and districts were trained for reporting malaria cases using the webpage of eCDS—MMS. *“Yes, I was trained and guided to report by software when I detect malaria cases”* (VHW, Binh Thuan Province).

### Case investigation and classification

From 1 January 2017 to 31 December 2021, amongst a total of 12,965 malaria cases, 62.3% (n = 8,071) were indigenous malaria cases. More than a quarter (n = 3541, 27.3%) were locally imported cases (i.e., from one commune, district or province to another one in Vietnam). Very few malaria cases were imported from overseas; 4.6% (n = 598); 3.8% (n = 487) from other GMS countries and 0.9% (n = 111) from countries outside of GMS. Over the five years, case classification was not done for 5.7% (n = 744) of all malaria cases detected as noted by missing that data in the secondary dataset (Table 1).

From January 2017 to December 2020, case investigation data was not available in the Excel database. Taking case classification as a proxy indicator for completeness of case investigation, it was found to be high (11,766/ 12,498, 94.1%) for that period. In 2021, almost all case investigations were completed (453/467, 97.0%), the majority in a timely manner (371/467, 79.4%) as per the secondary data analysis. The surveys supported these findings as almost all stakeholders/staff and FHSPs noted that there is a proper standard operation procedure (70/74, 94.6%) and case investigation forms (71/74, 95.9%) for case investigation, and 91.9% (34/37) of FHSPs confirmed that they follow the standard procedure (Additional file 5: Table S6).

In the surveys, enablers and barriers for timely case investigation was explored. About two thirds of the survey participants (51/72, 70.8%) mentioned that either an indigenous or imported malaria case detected triggered the case investigation through reporting of the detected cases to peripheral level health facilities (50/73, 68.5%). Major reasons for not performing or completing case investigation included that the malaria case couldn’t be found during the visit of centre staff (25/74, 35.7%) and the case has already been investigated by someone else (27/74, 38.6%). Difficulty contacting the case was identified as the common barrier for case investigation (28/70, 40%) (Additional file 5: Table S7).

In the FHSP survey, 19/38, 50% of FHSPs reported always being involved in case investigation. Furthermore, among the other half of FHSPs who sometimes or never involved in case investigation, (13/19), 68.4% were willing to participate in case investigation in the future with nearly a third of them (6/19, 31.6%) not answering this question. 34/36, 94.4% of programme management stakeholder/staff survey respondents also confirmed that there is specific person(s) in the malaria programme who is responsible for overseeing case investigations (Table 3). As per the survey, responsible FHSPs were trained in case investigation techniques (59/70, 84.3%) and they were provided with refresher trainings annually (22/67, 32.8%). 65/72, 90.3% of providers and stakeholders/staff mentioned that responsible FHSPs were supervised for case investigation monthly (21/72, 29.2%) and quarterly (22/72, 30.6%) (Table 3). *“I was trained and guided to report (malaria) cases by software (application), when I detect the cases. And also, to investigate case, classify locally or imported cases, and to investigate focus and respond to the disease focus*” (FHSP, Binh Thun Province).

**Table 3:** Staff involvement in, and training and supervision on case investigation and classification (Survey)

Information on case investigation	Health stakeholders/staff	FHSPs	Total
Involvement in case investigation, n (%)			
Yes, always	-na-	19 (50.0)	19 (50.0)
Yes, sometimes	-na-	16 (42.1)	16 (42.1)
No, never	-na-	3 (7.9)	3 (7.9)
Willingness to be involved in case investigation in future among FHSP who sometimes or never involved in case investigation* (n = 19), n (%)			
Yes	-na-	13 (68.4)	13 (68.4)
No	-na-	0 (0)	0 (0)
Missing	-na-	6(31.6)	6(31.6)
Responsible person for performing case investigation, n (%)			
Village health workers (VHWs) or equivalent	7 (19.4)	-na-	7 (19.4)
Others	29 (80.6)	-na-	29 (80.6)
Specific person in the malaria programme who is responsible for overseeing case investigations, n (%)			
Yes	34 (94.4)	-na-	34 (94.4)
No	2 (5.6)	-na-	2 (5.6)
Responsible person was trained in case investigation techniques, n (%)			
Yes	32 (91.4)	27 (77.1)	59 (84.3)
No	2 (5.7)	8 (22.9)	10 (14.3)
Don’t know	1 (2.9)	0 (0)	1 (1.4)
Frequency of the training relating to case investigation, n (%)			
Monthly	3 (8.6)	5 (15.6)	8 (11.9)
Quarterly	4 (11.4)	2 (6.3)	6 (9.0)
Yearly	20 (57.1)	2 (6.3)	22 (32.8)
Every second year	8 (22.9)	23 (71.9)	31 (46.3)
Being supervised for conducting case investigation, n (%)			
Yes	33 (94.3)	32 (86.5)	65 (90.3)
No	2 (5.7)	5 (13.5)	7 (9.7)
Frequency of supervision for personnel conducting case investigations, n (%)			
Monthly	6 (16.7)	15 (41.7)	21 (29.2)
Quarterly	18 (50.0)	4 (11.1)	22 (30.6)
Yearly	5 (13.9)	8 (22.2)	13 (18.1)
Others	7 (19.4)	9 (25.0)	16 (22.2)

In terms of detailed case investigation activities, many survey respondents (60/71, 84.5%) revealed that they always visit the index case household through a prior phone call appointment with the index case (51/73, 69.9%). Some FHSPs had to spend days travelling if the index case resided in hard-to-reach and remote areas, although the time taken to implement the case investigation only took a few minutes to one hour. *“Facilitator: For farthest, and how long does it take to investigate? FHSP: I have to travel for a day. Only for travelling. It takes about 20 min for a family of 5 people (to investigate). Depending on the household size. Some have 2 people, and some of them have 3 people.”* (A conversation in FGD with FHSP, Phu Yen Province).

According to the survey, if the index case does not show up during FHSP visit to household, they make a second visit (21/73, 28.8%), make a phone call for a second time appointment (42/73, 57.5%) or inform volunteers to make appointment with the index case for next visit (25/73, 34.3%) **(**Additional file 5: Table S8). As per conversations in FGDs with MMPs, community members also preferred to get an appointment before the visit so that they will be prepared for and ready to cooperate with the investigation. *“I am willing to provide information to commune health centre and agree to take blood smear test. I am just afraid of commune health centre staff come suddenly especially when I am not at home. However, I am willing to cooperate. Inform me one day in advance. Then in the next day, I will stay at home for investigation”* (MMP, Binh Thun Province).

According to the secondary dataset, over five years, nearly three quarters (9379/12965, 72.3%) of the malaria patients had a relevant travelling history within 14 days prior to diagnosis of malaria (eCDS—MMS 351/467, 75.2% and former paper-based reporting 9,028/12,498, 72.2%). The majority (eCDS—MMS 446/467, 95.5%) of malaria patients were directly observed of taking anti-malaria treatment by the health staff. However, supervision of antimalarial treatment and global positioning system (GPS) coordinates data were either collected sparingly or not at all in the former data collection system (2017–2020) (Table 4). Supporting the findings from secondary data analysis, the survey participants (48/72, 66.7%) mentioned that case investigation didn’t involve mapping the location of the index case via GPS**.**

**Table 4:** Detailed case investigation activities (Secondary data analysis)

	Paper-based system^a^	eCDS—MMS^b^	Total
Travelling history			
No	2566 (20.5%)	116 (24.8%)	2682 (20.7%)
Yes	9028 (72.2%)	351 (75.2%)	9379 (72.3%)
Missing	904 (7.2%)	0 (0.0%)	904 (7.0%)
Directly observed treatment of anti-malaria treatment by health staff			
No	60 (0.5%)	0 (0.0%)	60 (0.5%)
Yes	342 (2.7%)	446 (95.5%)	788 (6.1%)
Missing	12,096 (96.8%)	21 (4.5%)	12,117 (93.5%)
Global Positioning System (GPS) mapping			
No	-na-	-na-	-na-
Yes^c^	27 (0.2%)	-na-	27 (0.2%)
Missing	12,471 (99.8%)	467 (100.0%)	12,938 (99.8%)
Fever in family members of index case			
No	10,982 (87.9%)	0 (0.0%)	10,982 (84.7%)
Yes	462 (3.7%)	37 (7.9%)	499 (3.8%)
Missing	1054 (8.4%)	430 (92.1%)	1484 (11.4%)
Fever in neighbouring households of index case			
No	10,294 (82.4%)	0 (0.0%)	10,294 (79.4%)
Yes	1033 (8.3%)	144 (30.8%)	1177 (9.1%)
Missing	1171 (9.4%)	323 (69.2%)	1494 (11.5%)

^a^Microsoft Excel-based dataset for malaria case-based data collected via paper-based reporting system between January 2017 and December 2020

^b^Electronic Communicable Disease Surveillance system used in 2021

^c^No GPS coordinates, only the name of location was recorded

### Focus investigation and reactive case detection

RACD is a common activity in the focus investigation and response. The vast majority of stakeholders/staff and FHSPs in survey (62/73, 84.9%) mentioned that RACD was triggered after finding an indigenous case. According to the secondary data, in RACD, around 88% of fever screenings were done in household members (11,481/12,965, 88.6%) and in neighbouring households (11,471 / 12,965, 88.5%) from 2017 to 2021. Nearly all survey participants responded that household members of the index case and neighbouring households were always screened (67/73, 91.8%), and the majority responded that screening involved all household members in neighbouring households (50/73, 68.5%). Apart from the index household, participants used either neighbouring household (72/73, 98.6%), neighbouring household members (43/73, 58.9%) or radius distance from the index household (63/71, 88.7%) for RACD **(**Additional file 5: Table S9). *“The process is that when I detect a case of malaria, I will contribute to the next steps for that case. I test them (suspected cases) for malaria, and give them medicine, monitor them and guide them to take medicine. I also locate the area and take the blood samples from around the household and do communication”* (FHSP, Phu Yen Province). *“How many households do you test (for RACD)? About 15 households* (FHSP, Binh Thuan Province). Some FHSPs in FGDs suggested that RACD is done within 200 m radius of the index case household or nearest 15 to 30 households of the index household. *“For focus investigation, the blood smear is taken from around the household’s neighbours within 200 m”* (FHSP, Binh Thuan Province). Some FHSPs mentioned that they do RACD around the index household in the village without specifying a radius. *“In event that the case is in the village, the blood smear test (RACD) will be taken from the neighbours around that case”* FHSP, Binh Thuan Province). On some occasions, FHSPs mark a certain number of tests for RACD for a village where index case is detected. *“The ratio of blood smear test (for RACD) is 50 tests per village. We take blood from the household members who have contact with case directly, who work in the forest together with the case and adult with fever. No blood smear test for children is performed. Last year, when I detected a case of malaria, commune health centre asked me to take 50 blood smear tests. I followed the instruction from the District Health Centre.”* (FHSP, Phu Yen Province).

RDT and malaria microscopy was primarily used for RACD. In the secondary data analysis, it was found that only the eCDS—MMS database recorded the diagnostic method of malaria. Slightly more than two thirds (319/467, 68.3%) of the malaria patients from 1 January 2021 to 31 December 2021 were detected with both RDT and microscopy for their malaria diagnosis. *“If you go for RACD, what do you bring? RDT or blood smear? I bring both for testing to know the result right away and for treatment monitoring*” (FHSP, Phu Yen Province). Only two stakeholders/staff in survey mentioned that PCR was used for RACD. In RACD, the index case was treated as per the National Malaria Treatment Guidelines. Index household members are tested for malaria with microscopy first and neighbour household members are tested in the following days according to IDIs. *“The malaria patients take medicine and will do re-test after 3 days. Coordinate with VHWs to bring blood smear tests and medicine, if we detect new patients, we will treat them. It takes 1 h to take blood smear test for all household members, it is about 5–6 tests/ household. Do the rapid tests at households and bring the blood smear tests to commune health centre for examination at microscopy station. If there are no more malaria parasites, we continue to investigate with surrounding households. The investigation will be finished after from 4–5 days. Or on the third day, we investigate the surrounding areas”* (District level staff, Binh Thun Province).

According to stakeholders/staff surveyed (14/ 35, 40%), RACD is performed within 7 days after a positive case is recorded. Implementation of RACD seams feasible because VHWs accompanied them, and febrile cases cooperated the investigation according to FHSPs. Nevertheless, screening of malaria among afebrile people was still challenging according to FHSPs in FGDs. *“When we (FHSPs) go for testing, we go with VHWs and inform people about the location for RACD. People know it already. Those who have fever were very cooperative that made malaria testing very quick. However, those who don’t have fever were not willing to get tested and therefore, difficult to do so”* (FHSP, Binh Thuan Province). If some household members were missing, survey data showed that the RACD team scheduled another appointment for screening (44/73, 60.3%), or came back to the household later that day or on a subsequent day (31/73, 42.5%). The most common challenge in performing RACD in the community reported by survey respondents was inability to contact or find the index cases (23/73, 31.5% of respondents) **(**Additional file 5: Table S9). To execute RACD properly and timely, FHSPs and VHWs prefer to work as a team given the tasks could be shared among the team members that made saving time.

Apart from RACD, other focus response activities reported by survey respondents included raising awareness about malaria transmission (56/72, 77.8%) and prevention (61/72, 84.7%), providing additional vector control interventions (33/72, 45.8%), such as indoor residual spraying and entomological surveillance (19/72, 26.4%), and spot checking of mosquito breeding sites (10/72, 13.9%), and tailored interventions to the findings from case and focus investigations (57/69, 82.6%). Survey respondents believed that response activities were commenced within 1 (25/ 73, 34.3%) and 7 (21/73, 28.8%) days after the index case was notified. As there are many activities to be completed in focus response, it took a few days to complete all these activities. *“Last time it took about 3 days to spray and re-spray the remaining households (in the focus)”* (VHW, Phu Yen Province). A high proportion (29/35, 82.9%) of stakeholders/staff responded in the survey that current RASR activities are targeted to MMP, including forest-goers, and that current RASR activities are sufficient for targeting vivax malaria elimination (32/35, 91.4%) (Additional file 5: Table S10).

Although FHSPs know that insecticide spraying is required for active foci as per the guidelines, some FGD and IDI participants reported that it is not always completed due to limitation of budget. Furthermore, they believed that it is not always required given the transmission occurs in the forest and no vector in the village. *“No need to spray because all cases are in the forest, there is no more case in the village, no more vector”* (FHSP, Phu Yen Province).

## Discussion

This study comprehensively evaluated the current implementation of RASR and its feasibility of implementation in the context of malaria elimination programme in Vietnam. Overall, there were policy commitment, guidelines and procedures for implementation of each step of RASR in Vietnam such as case notification followed by case investigation and classification, and focus investigation and response. Completeness of case notification was very high (nearly 100%) in both the paper-based and eCDS—MMS reporting system; however, there were delays in notification while using the paper-based system. Either indigenous or imported malaria case detected triggered the case investigation through reporting of the detected cases to peripheral level health facilities. Date (and time) of case investigation could not be found in the paper-based reporting system (i.e., no data on completeness and timeliness of case investigation until December 2020). Nevertheless, case classification was taken as the proxy indicator and completeness of case investigation and classification was over 90%. After the eCDS—MMS being introduced at the start of 2021, the completeness and timeliness of case investigation were found to be high (453/467, 97.0% and 371/467, 79.4% in 2021, respectively). RACD is a common activity in the focus investigation and response and triggered after finding an indigenous case with fever screening of household members and in neighbouring household occurring in the vast majority of cases. Major barriers for RASR were contacting the patient for case investigation and focus investigation, and insufficient resources for transportation to and from the patient’s resident to execute RASR.

### Overall reactive surveillance and response strategies

In Vietnam, there are established guidelines and procedures of RASR including case notification, case investigation and focus investigation. Specific people to execute and supervise RASR were assigned at the commune, district and province levels. These staff were trained for RASR. Taken together, these findings point to the national policy commitments for RASR and ultimately for malaria elimination in Vietnam and the region. These policy commitments and efforts towards malaria elimination in Vietnam will ultimately contribute to the regional malaria elimination goal. At the field implementation level, most stakeholders/staff and FHSPs at provincial, district and commune levels were aware of these guidelines and procedure. Nevertheless, some FHSPs and VHWs could not recall the detailed procedures of RASR given the overwhelming assignment of multiple health programmes to them. As is typical in other GMS countries, almost all public health programmes in Vietnam tended to use FHSPs at field level when the work burden was high on FHSPs at commune and district levels. As a result, FHSPs could not implement quality activities in the field. Support such as incentive for RASR and transportation assistance are required to better implement RASR at the field level. Although there were limitations, FHSPs showed their willingness to follow guidelines and procedures. FHSPs were also willing to participate in RASR in the future as well. Using the available human resource and infrastructure, Vietnam could strengthen steps in RASR including case investigation and focus investigation with minimal additional use of budget that will ultimately return as an impact in malaria elimination programme.

### Case notification

Case notification of a malaria case is the RASR trigger. As per the secondary data analysis and quantitative survey, a high proportion of malaria case reporters possessed a mobile phone and email address, and the villages have high coverage of mobile phone network and internet access. Starting from 2021, Vietnam MoH deployed eCDS—MMS, an electronic reporting system for communicable diseases including malaria, which substantially improved the completeness and timeliness of case notification in Vietnam. Nevertheless, reporting via eCDS—MMS can only be successful if there is internet access in the rural forested areas where malaria cases are prevalent. In many rural areas in Vietnam, there was no mobile phone and internet network coverage outside the villages (in the forest). It caused delay in case notification for cases detected in the forest/ field site especially in active case detection executed by FHSPs and VHWs.

To overcome this issue and to achieve 100% timely notification of malaria cases, a new channel of connection such as satellite internet access could be established. However, establishment of an alternative connection network may require significant amount of technological investment, which in turn would require significant budget. Alternatively, FHSPs and VHWs could fill in the eCDS—MMS offline and travel to the location where there is internet access for synchronization of the application daily or on the alternative days. In this way, case notification within the required 48 h could be achieved. Nevertheless, time and effort of FHSPs and VHWs as well as budget for transportation have to be utilized for this approach. However, this approach could be feasible because the resources and cost would reduce over time along with the reduction of malaria cases in the latter phase of the malaria elimination programme.

### Case investigation and classification

When a malaria case is notified, FHSPs have to travel to the patient’s residence at least one time for case investigation. If they are unable to see the patient during the visit, they have to revisit which increases the time and effort of FHSPs on each case investigation. Major reasons for not performing case investigation are not meeting with the patient during the visit of FHSP and someone, probably other FHSPs, already performed case investigation. MMPs also mentioned that they preferred to get appointment first before FHSP case investigation visits so that they could be prepared for the case investigation. There were miscommunications between malaria patients and FHSPs as well as between FHSPs in the process of executing case investigation. To address this issue and for successful, timely and cost-effective implementation of case investigation, establishment of proper appointment system for case investigation is crucial.

Furthermore, FHSPs used “Form 1- Malaria case reporting and case investigation” [10] for case notification and case investigation starting from 2021. This new form is quite comprehensive, but it does not include recording of GPS coordinates from the index cases household. Sometimes, FHSPs experienced issues in tracing down the index cases because they could not contact the case by any means of communication. If the GPS coordinates are recorded when a malaria case is detected, it would facilitate case tracing and surveillance. To do this, FHSPs and VHWs need to be supported with GPS technology and training.

Currently, the developed eCDS—MMS webpage for the malaria elimination programme in Vietnam just focuses on case notification. There is no comprehensive application that includes the whole continuum of RASR including case notification with GPS coordinates, case investigation and classification, and focus investigation and response, and follow up monitoring of focus at day 30 and onward. This application needs to be developed and pilot tested in Vietnam to enhance systematic case investigation and followed up actions.

Over the 5 years of 2017–21, annual reported malaria cases declined from 4454 cases in 2019 to 467cases in 2021. Nevertheless, the majority of malaria cases in Vietnam were indigenous cases or locally imported cases within Vietnam (62.3% and 27.3% respectively). Interrupting local transmission in Vietnam will have the biggest impact in progressing towards 2030 malaria elimination target. This goal will be achieved with further investments in RASR for remaining reported malaria cases which will become less and less over the time.

### Focus investigation and reactive case detection

Although stakeholders and FHSPs believed that focus investigation happened with 7 days after detecting the index case currently, they appreciated team effort for focus investigation and response given many tasks of RACD and non-RACD to be undertaken for implementation of focus investigation in a timely manner. To sustain the comprehensive and timely execution of focus investigation and response, human resources and financial support needs to be reinforced at the district and provincial levels.

Like implementing RACD in other malaria endemic countries, there are no specified and detailed geospatial guidelines for RACD in Vietnam for people geographically proximal to the index case (hot spots) or among populations who share the same characteristic (hot pops). Although all RACD events screen index case household members, there was no specific demarcation for screening neighbouring households (hot spots). Furthermore, RACD in Vietnam did not screen co-workers and co-travellers and hot pops may be missed in malaria screening. Cambodia, a neighbour of Vietnam, is also a GMS country and tested the effectiveness of RACD among co-travellers and co-workers (hot pops) because case yield in traditional RACD among co-residents (hot spots) of the index case was very low. RACD on co-travellers and co-workers (hot pops) provided better results compared to traditional RACD (hot spots) among co-residents in Cambodia [7, 14, 15]. In light of this, Vietnam needs to develop and field test optimal RACD strategies that cover both hot spots and hot pops.

In RACD, RDT and microscopy were used for screening which may not detect low density *Plasmodium* spp. infections that can act as a hidden reservoir of infection [16–18]. The RACD would be only comprehensive when it can detect infections of any density. It is critical to detect and treat all infections in order to achieve malaria elimination in Vietnam and the GMS [17, 19, 20]. Detection of infections currently missed by RDT/microscopy through integration of more sensitive malaria diagnostic tool such as PCR centrally or at the level of health centre and district may improve surveillance of residual transmission and aid elimination of asymptomatic reservoirs. Acknowledging this requirement, NIMPE has started deploying PCR facilities to some malaria endemic provinces which is yet to be expanded to all the entire endemic provinces.

### Strengths and limitations of the study

This is the first study in Vietnam that has comprehensively evaluated national RASR strategy. The study has used diverse data collection methods for different participants ranging from grassroots level frontline providers to the national level policy makers and managers. The findings from each method were triangulated to enforce the validity of study findings. Secondary data analysis included nationwide malaria case-based data for five years (2017–2021), and national level stakeholders/staff were included in interviews. Therefore, the findings from these analyses are generalizable to Vietnam broadly. However, only available individual level data reported via Case Report to the Excel Database (paper-based reporting from 2017 to 2020) and eCDS-MMS (2021) were analysed although malaria cases reported via Monthly Aggregated Reports without individual level details are higher in number. Primary data collection of quantitative and qualitative data from field level stakeholders/staff and FHSPs was only completed in Phu Yen and Binh Thun Provinces due to COVID-19 interruptions, and additional qualitative assessment of RASR at these levels may be warranted in remaining provinces. Further, RASR strategies currently being implemented in other GMS countries should be evaluated according to their local context.

### Recommendations and conclusions

Overall, Vietnam has been implementing the RASR strategy embedded in the national malaria elimination agenda successfully. This is due to the higher-level political commitment and investments as well as the motivation and cooperation of FHSPs and VHWs at the field level. To further strengthen RASR in the last miles of malaria elimination in Vietnam, a set of recommendations has been provided (Table 5) to improve implementation of RASR in Vietnam. The improvement of RASR strategy will facilitate malaria elimination in Vietnam and contribute to regional malaria elimination goals.

**Table 5:** Summary of recommendations to improve RASR strategy in Vietnam

RASR step	Issues	Recommendations
Overall	Overwhelming assignment of multiple health programmes to commune and district levels FHSPs superposed by limited support to execute RASR	Provision of financial incentive and transportation assistance to FHSPs
Overall	No comprehensive mobile phone application for the whole continuum of RASR to be used by FHSPs	Development and deployment of a comprehensive RASR application that includes all the steps of RASR to FHSPs
Case notification	Delaying in notification of malaria cases detected outside the village where there was no mobile phone and internet network coverage	Filling in the eCDS—MMS offline and traveling (with the financial support of National Malaria Programme) to the location where there is internet access for synchronization of the application daily or on the alternative days
Case investigation	Miscommunications between malaria patient and FHSP as well as between FHSPs in case investigation	Establishment of proper appointment system for case investigation
Case investigation	Unable to trace down and contact the index cases	Inclusion of GPS data in the “Malaria case reporting and case investigation” Form, and supporting FHSPs and VHWs with GPS technology and training
Focus investigation	No specified and detailed guidelines for RACD	Development and field testing of optimal RACD strategies that cover both hot spots and hot pops
Focus investigation	Existing tools used in RACD only detect clinical malaria cases	Deployment of PCR technology to the district level health facilities

### Supplementary Information

**Additional file 1: **STROBE checklist of the manuscript.

**Additional file 2: **Secondary dataset of NIMPE downloaded on 18.3.2022 for analysis.

**Additional file 3: **Survey questionnaire 1 and 2.

**Additional file 4: **Topic guides for semi-structured in-depth interviews and focus group discussions.

**Additional file 5: **Supplementary tables and figures.

## Data Availability

The dataset supporting the conclusions of this article is included within the article and its additional file.

## Abbreviations

eCDS-MMS: Electronic communicable disease surveillance system—malaria management system
FGD: Focus group discussion
FHSP: Frontline health services providers
GMS: Greater Mekong Subregion
GPS: Global Positioning System
IDI: In-depth Interview
MMP: Mobile and migrant population
NIMPE: National institute of malariology, parasitology and entomology
PCR: Polymerase chain reaction
RACD: Reactive case detection
RASR: Reactive surveillance and response
RDT: Rapid diagnostic test
VHW: Village health workers
