# Supplementary material for: Performance and feasibility of reactive surveillance and response strategies for malaria elimination in Vietnam: a mixed-methods study
Source: Malar J. 2023 Aug 7;22:229. doi: 10.1186/s12936-023-04660-w (PMC10405448; doi:10.1186/s12936-023-04660-w)
Supplement: Supplementary file 1 — Additional file 1: STROBE checklist of the manuscript. [file 12936_2023_4660_MOESM1_ESM.docx]

**Performance and feasibility of reactive surveillance and response strategies for malaria elimination in Vietnam: a mixed-methods study**

**Additional file 1: STROBE Statement — checklist of items that should be included in reports of observational studies**

|  | Item No. | Recommendation | Page  No. | Relevant text from manuscript |
| --- | --- | --- | --- | --- |
| **Title and abstract** | 1 | (*a*) Indicate the study’s design with a commonly used term in the title or the abstract | 1 | a mixed-methods study |
|  |  | (*b*) Provide in the abstract an informative and balanced summary of what was done and what was found | 2 | - a mixed-methods study of 1) secondary data analysis of nationwide malaria case-based dataset from 1 January 2017 to 31 December 2021; 2) a quantitative survey, and 3) qualitative interviews and focus group discussions administered to central, provincial and district level stakeholders/staff and to the commune and community level front line health services providers was conducted. - In Vietnam, guidelines and procedures for implementation of each step of RARS are present. Completeness of case notification was very high in both the paper-based (12,463/12,498, 99.7%) and electronic (467/467, 100%) reporting systems; however, there were delays in notification while using the paper-based system (timely notification – 7,978/12,454, 64.1%). In 2021, the completeness (453/467, 97.0%) and timeliness (371/467, 79.4%) of case investigation were found to be high. In the reactive case detection (RACD), fever screening for malaria was done among 88.5% (11,477 / 12,965) of index case household members and 88.4% (11,462 / 12,965) of people in neighbouring households. |
| Introduction | | | |  |
| Background/ rationale | 2 | Explain the scientific background and rationale for the investigation being reported | 5 | The nationwide performance of Vietnam’s RARS has yet to be evaluated |
| Objectives | 3 | State specific objectives, including any prespecified hypotheses | 5 | This study aims to evaluate the performance and feasibility of the implementation of RARS in Vietnam in order to provide recommendations for improved RARS in terms of quality, effectiveness, and coverage in the context of existing national health system which will contribute to achieving malaria elimination goals in Vietnam and more broadly in the GMS. |
| Methods | | | |  |
| Study design | 4 | Present key elements of study design early in the paper | 5 | A mixed-methods study including secondary data analysis of a national malaria case-based dataset, quantitative survey, and qualitative interviews and focus group discussions |
| Setting | 5 | Describe the setting, locations, and relevant dates, including periods of recruitment, exposure, follow-up, and data collection | 6, 7 | - between 1 January 2017 to 31 December 2020 was extracted from the Excel Database (reported via paper-based forms) - between 1 January to 31 December 2021 was extracted from electronic Communicable Disease Surveillance System (ECDS) (reported using the application on the mobile device). - Phu Yen and Binh Thuan Provinces - between November 2021 and April 2022 - The survey, interview and FGD data collections approximately lasted for 45, 60 and 90 minutes respectively - All data collection happened in private locations where privacy was maintained. |
| Participants | 6 | (*a*) *Cohort study*—Give the eligibility criteria, and the sources and methods of selection of participants. Describe methods of follow-up  *Case-control study*—Give the eligibility criteria, and the sources and methods of case ascertainment and control selection. Give the rationale for the choice of cases and controls  *Cross-sectional study*—Give the eligibility criteria, and the sources and methods of selection of participants | 6, 7 | - extracted from the Excel Database (reported via paper-based forms) - extracted from electronic Communicable Disease Surveillance System (ECDS) - 36 health stakeholders/staff and 38 frontline health services providers (FHSPs)) - malaria program management staff - clinical service providers - Purposive sampling was used given the limitation in budget and travel restrictions due to COVID-19 at the time of data collection. - health stakeholders/staff and key experts at national and provincial levels (Phu Yen and Binh Thuan Provinces), FHSPs at district and commune levels and mobile and migrant population (MMPs) and forest goers in the community. A total of 70 people (34 FHSPs and 36 MMPs/forest goers, all participants were gender balanced) participated in the FGDs with approximately 4 to 5 participants per FGD in both provinces. |
|  |  | (*b*) *Cohort study*—For matched studies, give matching criteria and number of exposed and unexposed  *Case-control study*—For matched studies, give matching criteria and the number of controls per case |  | NA |
| Variables | 7 | Clearly define all outcomes, exposures, predictors, potential confounders, and effect modifiers. Give diagnostic criteria, if applicable | 7 | - Outcomes of interest were timeliness in case notification and investigation. - Exposure - NA |
| Data sources/ measurement | 8* | For each variable of interest, give sources of data and details of methods of assessment (measurement). Describe comparability of assessment methods if there is more than one group | 7 | - Timeliness in malaria notification and case investigation were derived from the dates of test result, date of reporting and date of case investigation |
| Bias | 9 | Describe any efforts to address potential sources of bias | 5,6 | A mixed-methods study including secondary data analysis of a national malaria case-based dataset, quantitative survey, and qualitative interviews and focus group discussions was conducted to assess malaria RARS in Vietnam.  Two stage sampling was applied by selecting the health facilities that implement RARS in the endemic areas in the two provinces followed by purposive selection of participants who have experiences in malaria control and elimination in the selected health facilities.  A total of 70 people (34 FHSPs and 36 MMPs/forest goers, all participants were gender balanced) participated in the FGDs with approximately 4 to 5 participants per FGD in both provinces. |
| Study size | 10 | Explain how the study size was arrived at | 6,7 | Survey: Two stage sampling was applied by selecting the health facilities that implement RARS in the endemic areas in the two provinces followed by purposive selection of participants who have experiences in malaria control and elimination in the selected health facilities.  Interviews: Interviewees were purposively recruited based on their roles managing malaria reporting data and to ensure representation from multiple organisational levels. |
| Quantitative variables | 11 | Explain how quantitative variables were handled in the analyses. If applicable, describe which groupings were chosen and why | 7, 8 | - NA |
| Statistical methods | 12 | (*a*) Describe all statistical methods, including those used to control for confounding | 7, 8 | - NA, descriptive analyses only |
|  |  | (*b*) Describe any methods used to examine subgroups and interactions |  | NA |
|  |  | (*c*) Explain how missing data were addressed | 8 | Derivation of completeness indicator for notification was straightforward; based on whether there was a date of reporting. Regarding completeness of case investigation, eCDS - MMS database contains date of case investigation, but in Excel database, case classification variable is taken as a proxy for completeness of case investigation. Timeliness in malaria notification and case investigation were derived from the dates of test result, date of reporting and date of case investigation. Descriptive analysis was done for reporters having a phone number or an email address, malaria parasite species notified, case investigation and case classification, foci investigation and RACD. |
|  |  | (*d*) *Cohort study*—If applicable, explain how loss to follow-up was addressed  *Case-control study*—If applicable, explain how matching of cases and controls was addressed  *Cross-sectional study*—If applicable, describe analytical methods taking account of sampling strategy |  | NA |
|  |  | (*e*) Describe any sensitivity analyses |  | NA |
| Results | | | | |
| Participants | 13* | (a) Report numbers of individuals at each stage of study—eg numbers potentially eligible, examined for eligibility, confirmed eligible, included in the study, completing follow-up, and analysed |  | NA |
|  |  | (b) Give reasons for non-participation at each stage |  | NA |
|  |  | (c) Consider use of a flow diagram |  | NA |
| Descriptive data | 14* | (a) Give characteristics of study participants (eg demographic, clinical, social) and information on exposures and potential confounders | 11,15,16 | - The majority of malaria patients worked in farms (6,530 / 12,965, 50.4%) and in the forest or were forest goers - Table 1,3,4 |
|  |  | (b) Indicate number of participants with missing data for each variable of interest | 12,13 | - Table 1: Missing |
|  |  | (c) *Cohort study*—Summarise follow-up time (eg, average and total amount) |  | NA |
| Outcome data | 15* | *Cohort study*—Report numbers of outcome events or summary measures over time |  | NA |
|  |  | *Case-control study—*Report numbers in each exposure category, or summary measures of exposure |  | NA |
|  |  | *Cross-sectional study—*Report numbers of outcome events or summary measures | 11,12,14,15,17,18,19,20 | - High levels of completeness were achieved with both paper-based (35/12,463, 99.7%, 2017-2020) and electronic reporting systems (ECDS introduced in 2021, 467/467, 100%). However, only 64.1% (7,978/12,498) of cases reported with paper-based reporting were notified in a timely manner (i.e., within 48 hours as per the Decision 4922), increasing to 99.5% (391/393) after the introduction of ECDS in 2021 - more than 90% of malaria case were notified within 24 hours (although the standard time of notification is 48 hours) after diagnosis - Almost all case investigations were completed (453/467, 97.0%), the majority in a timely manner (371/467, 79.4%) in 2021 - Enablers and barriers to timely malaria case notification were identified in the secondary data analysis, surveys and qualitative consultations. - the majority of respondents mentioned that their village has mobile phone network coverage (36/ 38, 94.7%) and internet access (30/38, 79.0%), and 83.3% (25/30) of participants confirmed that the quality of both mobile phone and internet connection was good. Nearly a third of FHSPs (11/38, 29.0%) received a phone call from suspected malaria patients about their illness - enablers and barriers for timely case investigation was explored - Major reasons for not performing or completing case investigation included that the malaria case couldn’t be found during the visit of centre staff (25/74, 35.7%) and the case has already been investigated by someone else (27/74, 38.6%). Difficultly contacting the case was identified as the common barrier for case investigation (28/70, 40%) - all FHSP (24/24), 100% were willing to continue participation in case investigation in the future. - Table 1,3,4,5 |
| Main results | 16 | (*a*) Give unadjusted estimates and, if applicable, confounder-adjusted estimates and their precision (eg, 95% confidence interval). Make clear which confounders were adjusted for and why they were included |  | NA |
|  |  | (*b*) Report category boundaries when continuous variables were categorized | 11,17 | - notified in a timely manner (i.e., within 48 hours as per the Decision 4922) - case investigations were required to be completed within 72 hours from the test result. Since then, it has changed to within 48 hours |
|  |  | (*c*) If relevant, consider translating estimates of relative risk into absolute risk for a meaningful time period |  | NA |
| Other analyses | 17 | Report other analyses done—eg analyses of subgroups and interactions, and sensitivity analyses |  | NA |
| Discussion | | | | |
| Key results | 18 | Summarise key results with reference to study objectives | 33 | - This study comprehensively evaluated the current implementation of RARS and its feasibility of implementation in the context of malaria elimination program in Vietnam - Completeness of case notification was very high - there were delays in notification while using the paper-based system - the completeness and timeliness of case investigation were found to be high |
| Limitations | 19 | Discuss limitations of the study, taking into account sources of potential bias or imprecision. Discuss both direction and magnitude of any potential bias | 39 | - primary data collection of quantitative and qualitative data from field level stakeholders/staff and FHSPs was only completed in Phu Yen and Binh Thun Provinces due to COVID-19 interruptions, and additional qualitative assessment of RARS at these levels may be warranted in remaining provinces. |
| Interpretation | 20 | Give a cautious overall interpretation of results considering objectives, limitations, multiplicity of analyses, results from similar studies, and other relevant evidence | 34, 35, 37,38,40 | - Specific people to execute and supervise RARS were assigned at the commune, district and province levels. These staff were trained for RARS activities. Taken together, these findings point to the national policy commitments for RARS and ultimately for malaria elimination in Vietnam and the region. These policy commitments and efforts towards malaria elimination in Vietnam will ultimately contribute to the regional malaria elimination goal. - Nevertheless, reporting via ECDS can only be successful if there is internet access in the rural forested areas where malaria cases are prevalent. In many rural areas in Vietnam, there was no mobile phone and internet network coverage outside the villages (in the forest). It caused delay in case notification for cases detected in the forest/ field site especially in active case detection executed by FHSPs and VHWs - There were miscommunications between malaria patients and FHSPs as well as between FHSPs in the process of executing case investigation. - Like implementing RACD in other malaria endemic countries (Win Han Oo et al, submitted), there is no specified and detailed guidelines for RACD in Vietnam for people geographically proximal to the index case (hot spots) or among populations who share the same characteristic (hot pops). - Cambodia, a neighbour of Vietnam, is also a GMS country and tested the effectiveness of RACD among co-travellers and co-workers because case yield in traditional RACD among co-residents (hot spots) of the index case was very low. RACD on co-travellers and co-workers (hot pops) provided better results compared to traditional RACD among co-residents in Cambodia [8, 15, 16]. In light of this, Vietnam needs to develop and field test optimal RACD strategies that cover both hot spots and hot pops. - In RACD, RDT and microscopy were used for screening which may not detect low density Plasmodium spp. infections that can act as a hidden reservoir of infection [17-19]. - It is critical to detect and treat all infections in order to achieve malaria elimination in Vietnam and the GMS [18, 20, 21]. - Overall, Vietnam has been implementing the RARS embedded in the national malaria elimination agenda successfully. This is due to the higher-level political commitment and investments as well as the motivation and cooperation of FHSPs and VHWs at the field level. |
| Generalisability | 21 | Discuss the generalisability (external validity) of the study results | 38, 39 | - The study has used diverse data collection methods for different participants ranging from grassroots level frontline providers to the national level policy makers and managers. The findings from each method were triangulated to enforce the validity of study findings. Secondary data analysis included nationwide malaria case-based data for five years (2017 – 2021), and national level stakeholders/staff were included in interviews. Therefore, the findings from these analyses are generalisable to Vietnam broadly. |
| Other information | | | | |
| Funding | 22 | Give the source of funding and the role of the funders for the present study and, if applicable, for the original study on which the present article is based | 42 | - United Nations Office for Project Services to all authors - Australian National Health and Medical Research Council and Australian Centre for Research Excellence in Malaria Elimination to FJIF - The Burnet Institute is funded by a Victorian State Government Operational Infrastructure Support grant |

*Give information separately for cases and controls in case-control studies and, if applicable, for exposed and unexposed groups in cohort and cross-sectional studies.

**Note:** An Explanation and Elaboration article discusses each checklist item and gives methodological background and published examples of transparent reporting. The STROBE checklist is best used in conjunction with this article (freely available on the Web sites of PLoS Medicine at http://www.plosmedicine.org/, Annals of Internal Medicine at http://www.annals.org/, and Epidemiology at http://www.epidem.com/). Information on the STROBE Initiative is available at www.strobe-statement.org.
