# Supplementary material for: Performance and feasibility of reactive surveillance and response strategies for malaria elimination in Vietnam: a mixed-methods study
Source: Malar J. 2023 Aug 7;22:229. doi: 10.1186/s12936-023-04660-w (PMC10405448; doi:10.1186/s12936-023-04660-w)
Supplement: Supplementary file 4 — Additional file 4: Topic guides for semi-structured in-depth interviews and focus group discussions. [file 12936_2023_4660_MOESM4_ESM.docx]

## Additional file 4: Topic guides for semi-structured interviews and focus group discussions

### Topic guide for semi-structured interview with malaria program stakeholders responsible for designing and overviewing malaria reactive surveillance and response policies and strategies (RARS)

This is the interview topic guide for the in-depth interview with the malaria program stakeholders responsible for designing and overviewing malaria reactive surveillance and response policies and strategies (RARS). It assesses their perceptions and practice of current malaria RARS policies and strategies, their opinions regarding feasibility and acceptability of these strategies, and suggestions for optimization of these strategies increasing their effectiveness in the current malaria elimination settings of the GMS.

This interview is expected to be conducted in about 1 – 1.5 hours. A breaktime of 10 – 15 minutes can be incorporated into the session.

| **Person** | **Responsibility** |
| --- | --- |
| Interviewer | Lead the interview and facilitate discussion to obtain enriched data using an ethical approach |
| Note taker | Note-taking, audio recording and supplementary facilitation |
| Translator | Translation of facilitator and participants discussion where necessary |

| **Information about the interview session** | | |
| --- | --- | --- |
| 1.1. | Name of the interviewer |  |
| 1.2. | Name of the note taker |  |
| 1.3. | Date (dd/mm/yyyy) |  |
| 1.4. | Start time |  |
| 1.5. | End time |  |
| 1.6. | Archival code |  |

| **Is it OK to audio-record this conversion? (Yes/No)** |  |
| --- | --- |

| **2. Brief explanation of scope of the study** | |
| --- | --- |
| *“Before starting our interview, I would like to briefly explain the scope of our study. Our study focuses mainly on the malaria reactive surveillance and response (RARS) strategies and activities in the GMS countries. Malaria RARS activities are part of the overall malaria surveillance activities and they include* ***all activities to be carried once a malaria positive case has been detected by a service provider****. They can include* ***malaria case notification, case investigations, reactive case detection, focus investigation, subsequent appropriate response activities and any others****. They may sometimes be referred to as CIFIR activities in some countries.*  *So, this interview will include questions about these malaria RARS activities and other related activities.”* | |
| **3. Background information of the participant** | |
| 3.1. | Gender of the participant ***(Just to be noted by the interviewer)*** |
| 3.2. | Could you briefly describe your organization and your department including its coverage/catchment area? |
| 3.3. | Could you briefly describe your current designation, including its level of representativeness? |
| 3.4. | How long have you been working in the current position? How long have you been working with malaria programs? Do you mind saying your completed years of age by now? |
| 3.5. | What are your roles and responsibilities relating the malaria elimination program, especially the malaria RARS activities? |

| **4. Existing malaria RARS strategies in the country and current practice** | |
| --- | --- |
| 4.1. | Could you describe and discuss the malaria RARS policy/policies and strategy/ strategies currently being implemented in your country?   1. What kind of activities are included?  - Case notification - Case investigation - Reactive case detection - Focus investigation - Response activities - Others (if any)  1. What are the standard guidelines and procedures for implementing these activities? 2. What is the standard/targeted time schedule for implementing these activities? *(e.g., China’s 1-3-7 strategy)* 3. What are the authoritative bodies for setting these policies and strategies in your countries? 4. Do you think the strategies in your country are different from those of your neighbouring countries, especially the GMS countries? How are they different? Why? |
| 4.2. | Do you think each and every activity of your strategies could be carried out according to your standard guidelines and procedures? Why?   - Case notification - Case investigation - Reactive case detection - Focus investigation - Response activities - Others (if any)  1. How about in terms of timeliness? Why? 2. How about in terms of completeness? Why? |

| **5. Feasibility of current malaria RARS strategies and activities** | |
| --- | --- |
| 5.1. | What are the challenges for strictly following the standard procedures and guidelines in implementation of these activities?   - Case notification - Case investigation - Reactive case detection - Focus investigation - Response activities - Others (if any)  1. What are the health system-related challenges?   In terms of:   - Political commitments - Human resources - Financial resources - Commodity resources - Technology - Any others   How can these challenges be overcome? What kinds of support will be needed?   1. What are the challenges related to existing infrastructure and sociocultural background of your country?   In terms of:   - Communication - Transportation - Political situation - Cultural background - Any others   How can these challenges be overcome? What kinds of support will be needed? |
| 5.2. | Do you think the time schedule of your current malaria RARS strategies is appropriate to be strictly followed in your current settings?   1. What are the challenges? 2. How can they be improved? What kinds of support will be needed? |
| 5.3. | What do you think are the strengths of your current malaria RARS strategies (probably over others)? Why?   1. What are the external factors favouring your current RARS strategies? Why?   ***(The same probing questions as above can be used.)*** |
| 5.4. | Do you think the malaria RARS strategies currently implemented in your country is a good one in terms of feasibility? Why? How can it be improved? |
| 5.5 | Has the COVID-19 pandemic had an impact on the ability to implement malaria RARS strategies in your country? If so, how? How can this impact be mitigated? |

| **6. Acceptability of current malaria RARS strategies and activities** | |
| --- | --- |
| 6.1. | Do you think the current malaria RARS strategies and activities are acceptable/ satisfactory for different malaria program stakeholders at different levels of implementation in different geographical areas? Why?   - Case notification - Case investigation - Reactive case detection - Focus investigation - Response activities - Others (if any)   At   - Current regional and national elimination settings - Policy making & strategic level - Managing level - Field implementation level - Beneficiary level - Any others  1. How can they be improved to make them more acceptable for the different stakeholders? |

| **7. Perceived effectiveness of current strategies** | |
| --- | --- |
| 7.1. | Do you think the current malaria RARS strategies are effective for your national and regional malaria elimination goals? And efficient? Why? |
| 7.2. | What do you think about the malaria incidence and prevalence in your country going in the past years?   1. Can you relate these changes to your current malaria RARS strategies? How? |
| 7.3. | How do you think your current malaria RARS strategies can be optimized to improve their effectiveness and efficiencies in your malaria elimination setting? What kinds of support will be needed? |
| 7.4 | How is the information collected during malaria case and foci investigations used for decision making in your malaria elimination settings? Are there ways that this information could be better utilised? |

| **8. Role of CHW in malaria RARS Strategies** | |
| --- | --- |
| 8.1. | What kind of community health workers are working with malaria elimination program in your countries? What are their regular malaria elimination activities? |
| 8.2. | What are the roles of these community health workers (CHWs) in the current malaria RARS strategies? How are they taking part in the different current malaria RARS activities?   - Case notification - Case investigation - Reactive case detection - Focus investigation - Response activities - Others (if any) |
| 8.3. | What are the challenges regarding the CHWs taking part in their malaria RARS activities? |
| 8.4. | How can the participation of CHWs in malaria RARS activities be optimized? (How can roles of CHWs in malaria RARS strategies be optimized?) What kinds of support will be needed? |

| **9. Optimization of malaria RARS strategies for MMPs** | |
| --- | --- |
| 9.1. | What do you think about the acceptability of your current malaria RARS strategies and activities from the perspectives of the beneficiaries in your country such as the community members or the villagers? Why? |
| 9.2. | Do you have any mobile and migrant populations in your country?   1. What kind of MMPs are there? Migrant workers? IDP? 2. Immigrant? Outmigrants? 3. How many of them are there? 4. What are the seasonal dynamics of the MMPs? |
| 9.3. | What are the challenges in implementing these malaria RARS activities among the MMPs? Why? |
| 9.4. | How can the malaria RARS strategies and activities be optimized for the MMPs? What kinds of support will be needed? |

| **10. Overall** | |
| --- | --- |
| 10.1. | On consideration of all these factors that we have discussed before, do you think the current malaria RARS strategies in your country are effective and efficient for achieving the national and regional malaria elimination goals? Why? |
| 10.2. | On consideration of all these factors that we have discussed before, do you think the current malaria RARS strategies in your country are best suited with your existing health system, infrastructural and socio-cultural backgrounds? Why? |
| 10.3. | How do you think the current malaria RARS strategies can be optimized, overcoming the existing barriers and improving their effectiveness in existing malaria elimination setting in your country? And in the GMS? |

| **Conclusion** | |
| --- | --- |
|  | This is the end of our interview.  Do you have any questions for me?  Thank you very much for your participation. |

| **End of session** |
| --- |

### Topic guide for focus group discussion with malaria program stakeholders responsible for managing or supervising field RARS activities

This is the topic guide to be used by the facilitator of the focus group discussion of malaria program stakeholders responsible for managing, coordinating and supervising the field level malaria RARS activities. It assesses their perceptions and practice of current malaria RARS policies and strategies, their opinions regarding feasibility and acceptability of these strategies, and suggestions for optimization of these strategies increasing their effectiveness in the current malaria elimination settings of the GMS.

This discussion is expected to be conducted in about 1.5 – 2 hours. A breaktime of 10 – 15 minutes can be incorporated into the session.

| **Person** | **Responsibility** |
| --- | --- |
| Facilitator | Lead the overall process and facilitate discussion to obtain enriched data using an ethical approach |
| Note taker | Note-taking, audio recording and supplementary facilitation |
| Translator | Translation of facilitator and participants discussion where necessary |

| **1. Information about the discussion session** | | |
| --- | --- | --- |
| 1.1. | Name of the facilitator |  |
| 1.2. | Name of the notetaker/s |  |
| 1.3. | Name of the translator/s |  |
| 1.4. | Date (dd/mm/yyyy) |  |
| 1.5. | Start time |  |
| 1.6. | End time |  |
| 1.7. | Archival code |  |

| **Is it OK to audio-record this conversion? (Yes/No)** |  |
| --- | --- |

| **2. Brief explanation of scope of the study** |
| --- |
| *“Before starting our discussion, I would like to briefly explain the scope of our study. Our study focuses mainly on the malaria reactive surveillance and response (RARS) strategies and activities in the GMS countries. Malaria RARS activities are part of the overall malaria surveillance activities and they include* ***all activities to be carried once a malaria positive case has been detected by a service provider****. They can include* ***malaria case notification, case investigations, reactive case detection, focus investigation, subsequent appropriate response activities and any others****. They may sometimes be referred to as CIFIR activities in some countries.*  *So, this interview will include questions about these malaria RARS activities and other related activities.”* |

| **3. Background information of the participants** | | | | | | |
| --- | --- | --- | --- | --- | --- | --- |
| 3.1. | Could you please briefly introduce yourself, including your age, sex, designation, department, organization and your level of representativeness? | | | | | |
|  | ***Age*** | ***Sex*** | ***Designation*** | ***Department*** | ***Organization*** | ***Remark*** |
| P1 |  |  |  |  |  |  |
| P2 |  |  |  |  |  |  |
| P3 |  |  |  |  |  |  |
| P4 |  |  |  |  |  |  |
| P5 |  |  |  |  |  |  |
| P6 |  |  |  |  |  |  |
| 3.2. | How long have you been working in the current position? How long have you been working with malaria programs? | | | | | |
| 3.3. | What are your roles and responsibilities relating the malaria elimination program (especially the malaria RARS activities)? | | | | | |

| **4. Existing malaria RARS strategies in the country and current practice** | |
| --- | --- |
| 4.1. | Could you describe and discuss what are the malaria RARS policies and strategies currently being implemented in your areas?   1. What kind of activities are included?  - Case notification - Case investigation - Reactive case detection - Focus investigation - Response activities - Others (if any)  1. What are the standard guidelines and procedures for implementing these activities? 2. What is the standard/targeted time schedule for implementing these activities? *(e.g., China’s 1-3-7 strategy)* |
| 4.2. | Do you think each and every activity of your strategies could be carried out according to your standard guidelines and procedures in your areas? Why?   - Case notification - Case investigation - Reactive case detection - Focus investigation - Response activities - Others (if any)  1. How about in terms of timeliness? Why? 2. How about in terms of completeness? Why? |
| 4.3. | Does anyone of you have to give services for malaria case finding and case management in the field?   1. Do you think malaria case finding and case management could be carried out according to your standard guidelines and procedures? Why? 2. How could they be improved? |

| **5. Feasibility of current malaria RARS strategies and activities** | |
| --- | --- |
| 5.1. | What are the challenges for strictly following the standard procedures and guidelines in implementation of these activities in your areas?   - Case notification - Case investigation - Reactive case detection - Focus investigation - Response activities - Others (if any)  1. What are the health system-related challenges?   In terms of:   - Political commitments - Human resources - Financial resources - Commodity resources - Technology - Any others   How can these challenges be overcome? What kinds of support will be needed?   1. What are the challenges related to existing background infrastructure of your areas?   In terms of:   - Communication - Transportation - Political situation - Cultural background - Any others   How can these challenges be overcome? What kinds of support will be needed?   1. Has the COVID-19 pandemic affected how RARS strategies and activities are carried out in your area? If so, how? |
| 5.2. | Do you think the time schedule of your current malaria RARS strategies is appropriate to be strictly followed in your current settings?   1. What are the challenges? 2. How can they be improved? What kinds of support will be needed? |
| 5.3. | What do you think are the strengths of your current malaria RARS strategies (probably over others)? Why?  What are the external factors favouring your current RARS strategies? Why?  ***(The same probing questions as above can be used.)*** |
| 5.4. | Do you think the malaria RARS strategies currently implemented in your area is a good one in terms of feasibility? Why? How can it be improved? |

| **6. Acceptability of current malaria RARS strategies and activities** | |
| --- | --- |
| 6.1. | Do you think the current malaria RARS strategies and its activities are acceptable/ satisfactory for different malaria program stakeholders at different levels of implementation in different geographical areas? Why?   - Case notification - Case investigation - Reactive case detection - Focus investigation - Response activities - Others (if any)   At   - Field implementation level like yours - Managerial level like your supervisors - Community level like the CHWs - Beneficiary level like the villagers including the MMPs  1. How can they be improved to make them more acceptable for the different stakeholders? |

| **7. Perceived effectiveness of current strategies** | |
| --- | --- |
| 7.1. | Do you think the current malaria RARS strategies are effective for achieving malaria elimination goals in your areas? And efficient? Why? |
| 7.2. | What do you think about the malaria incidence and prevalence in your areas going in the past years?   1. Can you relate these changes to your current malaria RARS strategies? How? |
|  | Is information gained from case and foci investigations currently used for decision making around response activities? If so, how? |
| 7.3. | How do you think your current malaria RARS strategies can be optimized to improve their effectiveness and efficiencies in your malaria elimination setting? What kinds of support will be needed? |

| **8. Role of CHW in malaria RARS Strategies** | |
| --- | --- |
| 8.1. | What kind of community health workers are working with malaria elimination program in your area? What are their regular malaria elimination activities? |
| 8.2. | What are the roles of these community health workers (CHWs) in the current malaria RARS strategies? How are they taking part in the different current malaria RARS activities?   - Case notification - Case investigation - Reactive case detection - Focus investigation - Response activities - Any others |
| 8.3. | What are the challenges regarding the CHWs taking part in the malaria RARS activities? |
| 8.4. | How can the participation of CHWs in malaria RARS activities be optimized? (How can roles of CHWs in malaria RARS strategies be optimized?) What kinds of support will be needed? |

| **9. Optimization of malaria RARS strategies for MMPs** | |
| --- | --- |
| 9.1. | What do you think about the acceptability of your current malaria RARS strategies and activities from the perspectives of the beneficiaries in your area such as the community members or the villagers? Why? |
| 9.2. | Do you have any mobile and migrant populations in your area?   1. What kind of MMPs are there? Migrant workers? IDP? 2. Immigrant? Outmigrants? 3. How many of them are there? 4. What are the seasonal dynamics of the MMPs? |
| 9.3. | What are the challenges in implementing these malaria RARS activities among the MMPs in your areas? Why? |
| 9.4. | How can the malaria RARS strategies and activities be optimized for the MMPs in your areas? What kinds of support will be needed? |

| **10. Overall** | |
| --- | --- |
| 10.1. | *On consideration of all these factors that we have discussed before,* do you think the current malaria RARS strategies are effective and efficient for achieving the malaria elimination goals in your areas and in your country? Why? |
| 10.2. | *On consideration of all these factors that we have discussed before*, do you think the current malaria RARS strategies are best suited with your existing health system, infrastructural and socio-cultural backgrounds of your areas? Why? |
| 10.3. | How do you think the current malaria RARS strategies can be optimized, overcoming the existing barriers and improving their effectiveness in existing malaria elimination setting in your areas and in your country? |

| **Conclusion** | |
| --- | --- |
|  | This is the end of our discussion.  Do you have any questions for me?  Thank you very much for your participation. |

| **End of session** |
| --- |

### Topic guide for focus group discussion with service providers who undertake malaria case detection and case management in the field and have to directly or indirectly take part in the field level malaria RARS activities

This is the topic guide to be used by the facilitator of the focus group discussion of service provider who usually do the malaria case detection and case management in the field and have to directly or indirectly take part in the field level malaria RARS activities. It assesses their perceptions and practice of current malaria RARS activities, their opinions regarding feasibility and acceptability of these activities, and suggestions for optimization of these activities increasing their effectiveness in the current malaria elimination settings of the GMS.

This discussion is expected to be conducted in about 1.5 – 2 hours. A breaktime of 10 – 15 minutes can be incorporated into the session.

| **Person** | **Responsibility** |
| --- | --- |
| Facilitator | Lead the overall process and facilitate discussion to obtain enriched data using an ethical approach |
| Note taker | Note-taking, audio recording and supplementary facilitation |
| Translator | Translation of facilitator and participants discussion where necessary |

| **1. Information about the discussion session** | | |
| --- | --- | --- |
| 1.1. | Name of the facilitator |  |
| 1.2. | Name of the notetaker/s |  |
| 1.3. | Name of the translator/s |  |
| 1.4. | Date (dd/mm/yyyy) |  |
| 1.5. | Start time |  |
| 1.6. | End time |  |
| 1.7. | Archival code |  |

| **Is it OK to audio-record this conversion? (Yes/No)** |  |
| --- | --- |

| **2. Brief explanation of scope of the study** |
| --- |
| *“Before starting our interview, I would like to briefly explain the scope of our study. Our study focuses mainly on the malaria reactive surveillance and response (RARS) strategies and activities in the GMS countries. Malaria RARS activities are part of the overall malaria surveillance activities and they include* ***all activities to be carried once a malaria positive case has been detected by a service provider****. They can include* ***malaria case notification, case investigations, reactive case detection, focus investigation, subsequent appropriate response activities and any others****. They may sometimes be referred to as CIFIR activities in some countries.*  *So, this interview will include questions about these malaria RARS activities and other related activities.”* |

| **3. Background information of the participants** | | | | | | |
| --- | --- | --- | --- | --- | --- | --- |
| 3.1. | Could you please briefly introduce yourself, including your age, sex, designation, department, organization and your level of representativeness? | | | | | |
|  | ***Age*** | ***Sex*** | ***Designation*** | ***Department*** | ***Organization*** | ***Remark*** |
| P1 |  |  |  |  |  |  |
| P2 |  |  |  |  |  |  |
| P3 |  |  |  |  |  |  |
| P4 |  |  |  |  |  |  |
| P5 |  |  |  |  |  |  |
| P6 |  |  |  |  |  |  |
| 3.2. | How long have you been working in the current position? How long have you been working with malaria programs? | | | | | |

| **4. Existing malaria activities in the country that you need to take part in** | |
| --- | --- |
| 4.1. | Could you describe and discuss what are the malaria services currently being provided in your area??   1. What kind of malaria services are provided?  - Malaria case finding - Malaria case management (Treatment & Refer) - Malaria prevention and BCC activities - Others (if any)  1. What are the standard guidelines and procedures for implementing these malaria service provision activities? 2. Do you think these malaria service provision activities could be carried out according to the standard guidelines and procedures? Why? How could they be improved? 3. What are your roles and responsibilities in RARS activities?  - Case notification - Case investigation - Reactive case detection - Focus investigation - Response activities - Others (if any)  1. What are the standard guidelines and procedures for implementing these RARS activities? 2. What is the standard/targeted time schedule for implementing these RARS activities? *(e.g., China’s 1-3-7 strategy)* |
| 4.2. | Do you think each and every activity could be carried out according to the standard guidelines and procedures in your areas? Why?   - Case notification - Case investigation - Reactive case detection - Focus investigation - Response activities - Others (if any)  1. How about in terms of timeliness? Why? 2. How about in terms of completeness? Why? |

| **5. Feasibility of current malaria RARS strategies and activities** | |
| --- | --- |
| 5.1. | What are the challenges for strictly following the standard procedures and guidelines in implementation of these activities in your areas?   - Case notification - Case investigation - Reactive case detection - Focus investigation - Response activities - Others (if any)  1. What are the challenges in implementing RARS activities from aspects of service providers?   In terms of:   - Human resources - Commodity resources (example: RDT, Phone, Motorcycle) - Technical capacity (example: Mobile phone literacy for reporting, Difficulty in implementing RARS activities) - Communication (Phone reporting) - Transportation - Financial resources - Political situations - Cultural background   How can these challenges be overcome? What kinds of support will be needed?   1. What are the challenges for implementing RARS activities from the aspect of villagers/beneficiaries? |
| 5.2. | Do you think the time schedule of current malaria RARS activities is appropriate to be strictly followed in your current settings?   1. What are the challenges? 2. How can they be improved? What kinds of support will be needed? |
| 5.3. | What do you think are the strengths of current malaria RARS activities (probably over others)? Why?  What are the external factors favouring your current RARS activities? Why?  ***(The same probing questions as above can be used.)*** |
| 5.4. | Do you think the malaria RARS activities currently implemented in your area is a good one in terms of feasibility? Why? How can it be improved? |

| **6. Acceptability of current malaria RARS strategies and activities** | |
| --- | --- |
| 6.1. | Do you think the current malaria RARS activities are acceptable/ satisfactory for different malaria program stakeholders at different levels of implementation in different geographical areas? Why?   - Case finding and management - Case notification - Case investigation - Reactive case detection - Focus investigation - Response activities - Others (if any)   At   - Community level like yours (example: CHW/Volunteers) - Managerial level like your supervisors - Beneficiary level like the villagers including the MMPs  1. How can they be improved to make them more acceptable for the different stakeholders? |

| **7. Perceived effectiveness of current strategies** | |
| --- | --- |
| 7.1. | Do you think the current malaria RARS activities are effective for achieving malaria elimination goals in your areas? And efficient? Why? |
| 7.2. | What do you think about the malaria incidence and prevalence in your areas going in the past years?   1. Can you relate these changes to your current malaria RARS activities? How? |
| 7.3. | How do you think your current malaria RARS activities can be optimized to improve their effectiveness and efficiencies in your malaria elimination setting? What kinds of support will be needed? |

***(Skip section 8 if all participants are CHWs)***

| **8. Role of CHW in malaria RARS Strategies** | |
| --- | --- |
| 8.1. | What kind of community health workers are working with malaria elimination program in your area? What are their regular malaria elimination activities? |
| 8.2. | What are the roles of these community health workers (CHWs) in the current malaria RARS activities? How are they taking part in the different current malaria RARS activities?   - Case finding and management - Case notification - Case investigation - Reactive case detection - Focus investigation - Response activities - Any others |
| 8.3. | What are the challenges regarding the CHWs taking part in the malaria RARS activities? |
| 8.4. | How can the participation of CHWs in malaria RARS activities be optimized? (How can roles of CHWs in malaria RARS activities be optimized?) What kinds of support will be needed? |

| **9. Optimization of malaria RARS strategies for MMPs** | |
| --- | --- |
| 9.1. | What do you think about the acceptability of your current malaria RARS activities from the perspectives of the beneficiaries in your area such as the community members or the villagers? Why? |
| 9.2. | Do you have any mobile and migrant populations in your area?   1. What kind of MMPs are there? Migrant workers? IDP? 2. Immigrant? Outmigrants? 3. How many of them are there? 4. What are the seasonal dynamics of the MMPs? |
| 9.3. | What are the challenges in implementing these malaria RARS activities among the MMPs in your areas? Why? |
| 9.4. | How can the malaria RARS activities be optimized for the MMPs in your areas? What kinds of support will be needed? |

| **10. Overall** | |
| --- | --- |
| 10.1. | *On consideration of all these factors that we have discussed before,* do you think the current malaria RARS activities are effective and efficient for achieving the malaria elimination goals in your areas and in your country? Why? |
| 10.2. | *On consideration of all these factors that we have discussed before*, do you think the current malaria RARS activities are best suited with your country’s contextual background? Why? |
| 10.3. | How do you think the current malaria RARS activities can be optimized, overcoming the existing barriers and improving their effectiveness in existing malaria elimination setting in your areas and in your country? |

| **Conclusion** | |
| --- | --- |
|  | This is the end of our discussion.  Do you have any questions for me?  Thank you very much for your participation. |

| **End of session** |
| --- |

### Topic guide for focus group discussion with mobile and migrant populations/ forest goers

This is the topic guide to be used by the facilitator of the focus group discussion of mobile and migrants who are at risks of contracting malaria. It assesses their perceptions and practice of current malaria RARS activities, their opinions regarding feasibility and acceptability of these activities, and suggestions for optimization of these activities increasing their effectiveness in the current malaria elimination settings of the GMS.

This discussion is expected to be conducted in about 1 – 1.5 hours. A breaktime of 10 – 15 minutes can be taken in between the session.

| **Person** | **Responsibility** |
| --- | --- |
| Facilitator | Lead the overall process and facilitate discussion to obtain enriched data using an ethical approach |
| Note taker | Note-taking, audio recording and supplementary facilitation |
| Translator | Translation of facilitator and participants discussion where necessary |

| **1. Information about the discussion session** | | |
| --- | --- | --- |
| 1.1. | Name of the facilitator |  |
| 1.2. | Name of the notetaker/s |  |
| 1.3. | Name of the translator/s |  |
| 1.4. | Date (dd/mm/yyyy) |  |
| 1.5. | Start time |  |
| 1.6. | End time |  |
| 1.7. | Archival code |  |

| **Is it OK to audio-record this conversion? (Yes/No)** |  |
| --- | --- |

| **2. Brief explanation of scope of the study** |
| --- |
| *“Before starting our interview, I would like to briefly explain the scope of our study. Our study focuses mainly on the malaria reactive surveillance and response (RARS) strategies and activities in the GMS countries. Malaria RARS activities are part of the overall malaria surveillance activities and they include* ***all activities to be carried once a malaria positive case has been detected by a service provider****. They can include* ***malaria case notification, case investigations, reactive case detection, focus investigation, subsequent appropriate response activities and any others****. They may sometimes be referred to as CIFIR activities in some countries.*  *So, this interview will include questions about these malaria RARS activities and other related activities.”* |

| **3. Background information of the participants** | | | | | |
| --- | --- | --- | --- | --- | --- |
| 3.1. | Could you please briefly introduce yourself, including your age, sex, village/ workplace, type and nature of migrant works? | | | | |
|  | ***Age*** | ***Sex*** | ***Village/ Worksite/ Camp*** | ***Type of MMP (To be noted by facilitator)*** | ***Remark*** |
| P1 |  |  |  |  |  |
| P2 |  |  |  |  |  |
| P3 |  |  |  |  |  |
| P4 |  |  |  |  |  |
| P5 |  |  |  |  |  |
| P6 |  |  |  |  |  |
| 3.2. | How long have you been working/ living in this village/ worksite/ camp? | | | | |
| 3.3. | Could you please describe your work nature?   1. What kinds of work do you do? 2. Immigrant? Outmigrant? 3. How many people like you in your village/ worksite/ camp? 4. What are the seasonal dynamics of the MMPs? | | | | |

| **4. Perception and experience on malaria and RARS activities** | |
| --- | --- |
| 4.1. | Could you please briefly describe current malaria situation in your area? (Common village places and MMP’s workplace)   1. Could you please describe and discuss what kinds of service provider are providing malaria service in your area? 2. What are the malaria services currently being received in your area?  - Malaria testing - Malaria case management (Treatment & Refer) - Malaria prevention and BCC services - Others (if any)  1. Could you please describe your experience in receiving malaria case management and outcomes as a malaria suspect or patient or family and friend or neighbours or co-workers? |
| 4.2. | Could you please describe what kinds of activity are carried on by the service provider when a malaria positive patient has been detected, apart from malaria testing, case management and prevention and BCC activities?   - Case notification - Case investigation - Reactive case detection - Focus investigation - Response activities   How are these activities conducted by the service provider? (Mechanism of implementing these activities)   - - Common village places   - MMP’s workplaces |

| **Brief explanation of the mechanisms of current malaria RARS activities in the area investigated by the facilitator.**  *“As explain earlier, malaria reactive surveillance and response (RARS) activities* ***are to be carried out once a malaria positive case has been detected by a service provider****. These activities can include* ***malaria case notification (mechanism), case investigations (mechanism), reactive case detection (mechanism), focus investigation (mechanism), subsequent appropriate response activities and any others****.* |
| --- |

| **5. Acceptability and Feasibility of current malaria RARS strategies and activities** | |
| --- | --- |
| 5.1. | Could you please describe how do you receive malaria testing and treatment services from the service provider if you suspect malaria?   - Do you think you are acceptable/ satisfactory in receiving these services from the service provider? Why?   Does the service provider pay a schedule visit for malaria testing and treatment services to your workplace and if yes, how frequent?   - Do you think the current service provider’s scheduled visit for malaria testing and treatment service meets your needs? Why? |
| 5.2. | Could you please describe how you cooperate in malaria RARS activities?   1. What are the challenges as MMPs in cooperating the case notification activity?  - Any sensitive issues - Others (if any)  1. What are the challenges as MMPs in cooperating the case investigation activity?  - Any sensitive issues or questions - Recalling events - Others (if any)  1. What are the challenges as MMP in cooperating the reactive case detection (RACD) activity?  - Any missing co-workers - Security issues - Others (if any)  1. What are the challenges as MMPs in cooperating the focus investigation activity? 2. What are the challenges as MMPs in cooperating the response activities?  - IRS - BCC - LLIN - Larva source management - Others (if any)   How can these challenges be overcome? What kinds of support will be needed? |
| 5.3. | What are the challenges in cooperating the RARS activities from the aspects of MMPs?  In terms of:   - Human resources - Communication (Language, mobile phone) - Transportation - Financial resources - Political situations - Cultural background - Cooperation opportunities - Others (if any)   How can these challenges be overcome? What kinds of support will be needed? |
| 5.4. | Do you think the time schedule of current malaria RARS activities is appropriate in your current settings?   1. What are the challenges? 2. How can they be improved? What kinds of support will be needed? |
| 5.5. | Do you think the malaria RARS activities currently implemented in your area is a good one in terms of feasibility? Why? How can it be improved? |

| **6. Overall** | |
| --- | --- |
| 10.1. | *On consideration of all these factors that we have discussed before,* do you think the current malaria RARS activities are acceptable/ convenient for you? Why? |
| 10.2. | *On consideration of all these factors that we have discussed before*, do you think the current malaria RARS activities are best suited with your country’s contextual background? Why? |
| 10.3. | How do you think the current malaria RARS activities can be optimized, overcoming the existing barriers and improving their effectiveness in current setting? |

| **Conclusion** | |
| --- | --- |
|  | This is the end of our discussion.  Do you have any questions for me?  Thank you very much for your participation. |

| **End of session** |
| --- |
