# Supplementary material for: Performance and feasibility of reactive surveillance and response strategies for malaria elimination in Vietnam: a mixed-methods study
Source: Malar J. 2023 Aug 7;22:229. doi: 10.1186/s12936-023-04660-w (PMC10405448; doi:10.1186/s12936-023-04660-w)
Supplement: Supplementary file 5 — Additional file 5: Supplementary tables and figures. [file 12936_2023_4660_MOESM5_ESM.docx]

# **Additional file 5: Supplementary tables**

## **Supplementary Table 1: Background information of study participants in the survey**

| **Characteristic** | **Health stakeholders/ staff** | **Frontline health services providers (FHSPs)** | **Total** |
| --- | --- | --- | --- |
| Number of respondents interviewed | 36 | 38 | 74 |
| Completed age in years, mean (Standard Deviation (SD)) | 42.3 (10.4) | 41.3 (10.0) | 41.8 (10.1) |
| Gender, n (%) | | |  |
| Male | 26 (72.2) | 14 (36.8) | 40 (54.1) |
| Female | 10 (27.8) | 24 (63.2) | 34 (46.0) |
| Highest education level, n (%) | | | |
| No formal education | 1 (2.8) | 0 (0) | 1 (1.4) |
| Primary school level | 0 (0) | 1 (2.6) | 1 (1.4) |
| Secondary school level | 0 (0) | 4 (10.5) | 4 (5.4) |
| High school level | 0 (0) | 7 (18.4) | 7 (9.5) |
| Degree holder | 9 (25.0) | 6 (15.8) | 15 (20.3) |
| Others | 26 (72.2) | 20 (52.6) | 46 (62.2) |
| Current role of respondent, n (%) | | | |
| Malaria diagnosis, prevention & control | 14 (38.9) | -na- | 14 (18.9) |
| Malaria research & surveillance | 10 (27.8) | -na- | 10 (13.5) |
| Management role | 9 (25.0) | -na- | 9 (12.2) |
| Medical doctor | -na- | 3 (7.9) | 3 (4.1) |
| Nurse | -na- | 3 (7.9) | 3 (4.1) |
| Midwife | -na- | 1 (2.6) | 1 (1.4) |
| Village health worker | -na- | 14 (36.8) | 14 (18.9) |
| Health centre staff | -na- | 10 (26.3) | 10 (13.5) |
| Others | 3 (8.3) | 7 (18.4) | 10 (13.5) |
| Years of service in current role, mean (SD) | 15.9 (11.1) | 14.1 (8.9) | 15.0 (10.0) |

## **Supplementary Table 2: Participants in qualitative data collection of Focus Group Discussions (FGDs) and semi-structured interviews**

| **Methods** | **Province** | **# Participant** | **Remark** |
| --- | --- | --- | --- |
| Semi-structured interview with health stakeholders/ staff | Phu Yen | 16 | Interview with district and provincial level staff |
| FGD with FHSPs | Phu Yen | 16 | 4 participants / FGD x 4 FGDs |
| FGD with mobile migrant populations (MMPs) | Phu Yen | 16 | 4 participants / FGD x 4 FGDs |
| Semi-structured interview with health stakeholders/ staff | Binh Thuan | 12 | Interview with district and provincial level staff |
| FGD with FHSPs | Binh Thuan | 18 | 4-5 participants / FGD x 4 FGD |
| FGD with MMPs | Binh Thuan | 20 | 5 participants / FGD x 4 FGD |
| Total | 2 Provinces | 98 | 28 interviews and 16 FGDs |

## **Supplementary Table 3: General information on reactive surveillance and response strategies (RARS) approaches and malaria elimination programme (Survey)**

| **Information on RARS approaches and malaria elimination programme** | **Health stakeholders/ staff** | **FHSPs** | **Total** |
| --- | --- | --- | --- |
| Presence of time-bound strategy for case reporting, case investigation and response activities, n (%) | | | |
| No | 1 (2.8) | 2 (5.7) | 3 (4.2) |
| Yes, 1-3-7 approach | 16 (44.4) | 18 (51.4) | 34 (47.9) |
| Yes, other approach | 19 (52.8) | 15 (42.9) | 34 (47.9) |
| Place where the time-bound strategy for case reporting, case investigation and response activities are applied, n (%) | | | |
| Areas in elimination phase | 11 (31.4) | 8 (22.2) | 19 (26.8) |
| All areas | 20 (57.1) | 21 (58.3) | 41 (57.8) |
| Others | 4 (11.4) | 4 (11.1) | 8 (11.3) |
| Don’t know | 0 (0) | 3 (8.3) | 3 (4.2) |

## **Supplementary Table 4: Malaria cases reported in Vietnam from January 2017 to December 2021 (Secondary data analysis)**

| **Malaria parasite species** | **2017** | **2018** | **2019** | **2020** | **2021** |
| --- | --- | --- | --- | --- | --- |
| *P. falciparum* | 1,524 (63.4%) | 2,669 (63.2%) | 2,994 (67.2%) | 813 (57.4%) | 209 (44.8%) |
| *P. vivax* | 845 (35.2%) | 1,470 (34.8%) | 1,422 (31.9%) | 588 (41.5%) | 254 (54.4%) |
| *P. malarie* | 6 (0.2%) | 10 (0.2%) | 7 (0.2%) | 3 (0.2%) | 2 (0.4%) |
| *P. ovale* | 0 (0.0%) | 2 (0.0%) | 3 (0.1%) | 3 (0.2%) | 0 (0.0%) |
| Mixed infection | 27 (1.1%) | 74 (1.8%) | 28 (0.6%) | 10 (0.7%) | 2 (0.4%) |
| Total | 2,402 | 4,225 | 4,454 | 1,417 | 467 |

## **Supplementary Table 5: Malaria testing and reporting from the frontline health services providers (FHSP) to the responsible stakeholders/staff (Survey)**

| **Information related to malaria testing and reporting** | **Health stakeholders/ staff** | **FHSPs** | **Total** |
| --- | --- | --- | --- |
| **Performance on malaria service provision by the FHSPs** | | | |
| Number of malaria diagnostic tests performed in a month by the provider, mean (Standard Deviation (SD)) | -na- | 75.8 (77.7) | 75.8 (77.7) |
| Number of days in a month performing malaria diagnosis using RDT by the provider, mean (SD) | -na- | 16.3 (10.3) | 16.3 (10.3) |
| **First method to report positive malaria cases, n (%)** | | | |
| Paper-based reporting | 8 (24.2) | 12 (32.4) | 20 (28.6) |
| Electronic reporting system | 24 (72.7) | 10 (27.0) | 34 (48.6) |
| Telephone call | 8 (24.2) | 19 (51.4) | 27 (38.6) |
| Messaging program | 3 (9.1) | 0 (0) | 3 (4.3) |
| **Frequency of reporting malaria cases within 24 hours of diagnosis, n (%)** | | | |
| Never | 0 (0) | 1 (2.7) | 1 (1.4) |
| Occasionally (< 20%) | 4 (11.1) | 4 (10.8) | 8 (11.0) |
| Sometimes (20%-50%) | 1 (2.8) | 1 (2.7) | 2 (2.7) |
| More often (50%-75%) | 2 (5.6) | 3 (8.1) | 5 (6.9) |
| Usually (75%-90%) | 9 (25.0) | 6 (16.2) | 15 (20.6) |
| Nearly always (> 90%) | 20 (55.6) | 22 (59.5) | 42 (57.5) |

## **Supplementary Table 6: Standard operation procedure of case investigation (Survey)**

| **Information on case investigation** | **Health stakeholders/ staff** | **FHSPs** | **Total** |
| --- | --- | --- | --- |
| Presence of standard operation procedure for case investigation, n (%) | | | |
| Yes | 36 (100.0) | 34 (89.5) | 70 (94.6) |
| No | 0 (0) | 4 (10.5) | 4 (5.4) |
| Follow the standard operation procedure for case investigation, n (%) | | | |
| Yes | -na- | 34 (91.9) | 34 (91.9) |
| No | -na- | 3 (8.1) | 3 (8.1) |
| Specific form for case investigation, n (%) | | | |
| Yes | 36 (100.0) | 35 (92.1) | 71 (95.9) |
| No | 0 (0) | 3 (7.9) | 3 (4.1) |

## **Supplementary Table 7: Case investigation, its completeness and timeliness (Survey)**

| **Information on case investigation** | **Health stakeholders/ staff** | **FHSPs** | **Total** |
| --- | --- | --- | --- |
| **Trigger for a case investigation, n (%)** | | | |
| Case reported to national level | 10 (27.8) | 1 (2.7) | 11 (15.1) |
| Case reported to peripheral level | 17 (47.2) | 33 (89.2) | 50 (68.5) |
| Others | 9 (25.0) | 3 (8.1) | 12 (16.4) |
| **Policy for conducting a case investigation, n (%)** | | | |
| All indigenous and imported cases | 24 (68.6) | 27 (73.0) | 51 (70.8) |
| Indigenous cases only | 9 (25.7) | 8 (21.6) | 17 (23.6) |
| Imported cases only | 1 (2.9) | 0 (0) | 1 (1.4) |
| Others | 1 (2.9) | 0 (0) | 1 (1.4) |
| Don’t know | 0 (0) | 2 (5.4) | 2 (2.8) |
| **Extent of case investigation completed for positive malaria cases, n (%)** | | | |
| For all cases (100%) | 27 (77.1) | 20 (52.6) | 47 (64.4) |
| For <20% of cases | 0 (0) | 0 (0) | 0 (0) |
| Between 20% and 50% of cases | 2 (5.7) | 0 (0) | 2 (2.7) |
| Between 50% and 75% of cases | 0 | 4 (10.5) | 4 (5.5) |
| More than 75% of cases but less than 100% | 6 (17.1) | 14 (36.8) | 20 (27.4) |
| **Main reasons for cases that were not investigated, n (%)** | | | |
| Case was an imported case | 1 (2.8) | 8 (23.5) | 9 (12.9) |
| Case was outside of the district of the person investigating | 1 (2.8) | 10 (29.4) | 11 (15.7) |
| The person could not be found | 7 (19.4) | 18 (52.9) | 25 (35.7) |
| Not enough staff/resources | 2 (5.6) | 2 (5.9) | 4 (5.7) |
| Case was a daily cross-border case | 3 (8.3) | 5 (14.7) | 8 (11.4) |
| Not applicable – every case is investigated | 18 (50.0) | 9 (26.5) | 27 (38.6) |
| Others | 4 (11.1) | 2 (5.9) | 6 (8.6) |
| **How soon was case investigation initiated after a positive case is recorded, n (%)** | | | |
| Within 24 hours | 3 (8.3) | 12 (31.6) | 15 (20.3) |
| Within 48 hours | 16 (44.4) | 5 (13.2) | 21 (28.4) |
| Within 72 hours | 14 (38.9) | 14 (36.8) | 28 (37.8) |
| Within one week | 1 (2.8) | 4 (10.5) | 5 (6.8) |
| Within one month | 2 (5.6) | 3 (7.9) | 5 (6.8) |
| **Challenges in conducting case investigation, n (%)** | | | |
| No challenge | 10 (27.8) | 9 (26.5) | 19 (27.1) |
| Language barrier | 3 (8.3) | 1 (2.9) | 4 (5.7) |
| Insufficient information from patient/uncooperative patient | 3 (8.3) | 10 (29.4) | 13 (18.6) |
| Difficult or unable to contact/find patient | 14 (38.9) | 14 (41.2) | 28 (40.0) |
| Insufficient funding | 3 (8.3) | 2 (5.9) | 5 (7.1) |
| Insufficient manpower | 4 (11.1) | 1 (2.9) | 5 (7.1) |
| Weather and transportation difficulties | 2 (5.6) | 8 (23.5) | 10 (14.3) |

## **Supplementary Table 8: Detailed case investigation activities (Survey)**

| **Information on case investigation** | **Health stakeholders/ staff** | **FHSPs** | **Total** |
| --- | --- | --- | --- |
| **Visiting the index case during case investigation, n (%)** | | | |
| Yes, always | 25 (73.5) | 35 (94.6) | 60 (84.5) |
| No, never | 1 (2.9) | 1 (2.7) | 2 (2.8) |
| Yes, sometimes | 8 (23.5) | 1 (2.7) | 9 (12.7) |
| **Method to make appointment with the index case to do a case investigation, n (%)** | | | |
| Telephone the index case | 28 (80.0) | 23 (60.5) | 51 (69.9) |
| No prior communication | 4 (11.4) | 6 (15.8) | 10 (13.7) |
| Others | 3 (8.6) | 9 (23.7) | 12 (16.4) |
| **What was done if the index case was not home when they visited, n (%)** | | | |
| Visit a second time: later that day or on the day after | 8 (22.9) | 13 (34.2) | 21 (28.8) |
| Telephone to schedule an appointment | 18 (51.4) | 24 (63.2) | 42 (57.5) |
| Mark the case as ‘imported’ | 0 (0) | 0 (0) | 0 (0) |
| Mark the case as ‘not found’ | 1 (2.9) | 1 (2.6) | 2 (2.7) |
| Do not re-visit the index case | 0 (0) | 0 (0) | 0 (0) |
| Inform volunteers to make appointment with the case | 13 (37.1) | 12 (31.6) | 25 (34.3) |
| Others | 3 (8.6) | 5 (13.2) | 8 (11.0) |
| **Case investigation involved checking on malaria preventive measures used by the index case, n (%)** | | | |
| Yes | 35 (97.2) | 37 (100.0) | 72 (98.6) |
| No | 1 (2.8) | 0 (0) | 1 (1.4) |
| **Supervised treatment conducted for positive cases, n (%)** | | | |
| Yes | 30 (85.7) | 34 (89.5) | 64 (87.7) |
| No | 5 (14.3) | 4 (10.5) | 9 (12.3) |
| **Follow-up on adherence to treatment conducted for each case, n (%)** | | | |
| Yes | 33 (91.7) | 35 (94.6) | 68 (93.2) |
| No | 3 (8.3) | 2 (5.4) | 5 (6.9) |
| **Case investigation involved educating the index case on malaria risk factors and prevention, n (%)** | | | |
| Yes | 35 (97.2) | 36 (97.3) | 71 (97.3) |
| No | 1 (2.8) | 1 (2.7) | 2 (2.7) |
| **Case investigation involved mapping the location of the index case, n (%)** | | | |
| Yes | 13 (36.1) | 10 (27.8) | 23 (31.9) |
| No | 22 (61.1) | 26 (72.2) | 48 (66.7) |
| Don’t know | 1 (2.8) | 0 (0) | 1 (1.4) |
| **Programme collected data and reported on intra-country importation of cases, n (%)** | | | |
| Yes | 35 (97.2) | 35 (92.1) | 70 (94.6) |
| No | 1 (2.8) | 3 (7.9) | 4 (5.4) |
| **How imported case was defined, n (%)** | | | |
| Case originating in another country | 4 (11.4) | 1 (2.7) | 5 (6.9) |
| Case occurring within the country but from a different province, district or other administrative units | 20 (57.1) | 29 (78.4) | 49 (68.1) |
| All of the above | 7 (20.0) | 2 (5.4) | 9 (12.5) |
| Others | 4 (11.4) | 5 (13.5) | 9 (12.5) |

## **Supplementary Table 9: Foci investigation including reactive case detection (RACD) (Survey)**

| **Information on foci investigation and reactive case detection** | **Health stakeholders/ staff** | **FHSPs** | **Total** |
| --- | --- | --- | --- |
| **Trigger for RACD, n (%) by type of cases detected** | | | |
| Every indigenous case | 31 (88.6) | 31 (81.6) | 62 (84.9) |
| All imported cases irrespective of duration of stay | 0 (0) | 5 (13.2) | 5 (6.9) |
| Imported cases if they have stayed more than a certain number of days in country | 0 (0) | 0 (0) | 0 (0) |
| Every indigenous case and all imported cases irrespective of duration of stay | 4 (11.4) | 2 (5.3) | 6 (8.2) |
| **Trigger for RACD, n (%) by number of cases detected** | | | |
| Single confirmed case | 29 (82.9) | 32 (86.5) | 61 (84.7) |
| >1 confirmed case within a specified radius | 2 (5.7) | 2 (5.4) | 4 (5.6) |
| Other threshold of confirmed cases | 4 (11.4) | 1 (2.7) | 5 (6.9) |
| Don’t know | 0 (0) | 2 (5.4) | 2 (2.8) |
| **How soon** **foci investigation is initiated after a positive case is recorded, n (%)** | | | |
| Within 24 hours | 3 (8.6) | -na- | 3 (8.6) |
| Within 48 hours | 4 (11.4) | -na- | 4 (11.4) |
| Within 72 hours | 10 (28.6) | -na- | 10 (28.6) |
| Within 7 days | 14 (40.0) | -na- | 14 (40.0) |
| Within 14 days | 3 (8.6) | -na- | 3 (8.6) |
| Within 28 days | 1 (2.9) | -na- | 1 (2.9) |
| **Screening a minimum number of households around a positive index case, n (%)** | | | |
| Yes | 35 (100.0) | 37 (97.4) | 72 (98.6) |
| No | 0 (0) | 1 (2.6) | 1 (1.4) |
| **Screening a minimum number of people around a positive index case, n (%)** | | | |
| Yes | 23 (65.7) | 20 (52.6) | 43 (58.9) |
| No | 12 (34.3) | 18 (47.4) | 30 (41.1) |
| **Screening within a minimum geographical radius around a positive index case, n (%)** | | | |
| Yes | 30 (88.2) | 33 (89.2) | 63 (88.7) |
| No | 4 (11.8) | 4 (10.8) | 8 (11.3) |
| **Diagnostic method used when conducting RACD, n (%)** | | | |
| Microscopy | 32 (91.4) | 24 (64.9) | 56 (77.8) |
| Rapid diagnostic Test (RDT) | 28 (80.0) | 32 (86.5) | 60 (83.3) |
| Polymerase chain reaction (PCR) | 2 (5.7) | 0 (0) | 2 (2.8) |
| Clinical diagnosis | 4 (11.4) | 4 (10.8) | 8 (11.1) |
| Serology | 1 (2.9) | 0 (0) | 1 (1.4) |
| **Frequency of screening household members of the index case, n (%)** | | | |
| Always | 32 (91.4) | 35 (92.1) | 67 (91.8) |
| Never | 2 (5.7) | 1 (2.6) | 3 (4.1) |
| Sometimes | 1 (2.9) | 2 (5.3) | 3 (4.1) |
| **Frequency of screening neighbors of the index case in the community, n (%)** | | | |
| Always | 32 (91.4) | 35 (92.1) | 67 (91.8) |
| Sometimes | 3 (8.6) | 3 (7.9) | 6 (8.2) |
| Never | 0 (0) | 0 (0) | 0 (0) |
| **Type of neighbors screened, n (%)** | | | |
| Febrile neighbors only | 9 (25.7) | 14 (36.8) | 23 (31.5) |
| All neighbors | 26 (74.3) | 24 (63.2) | 50 (68.5) |
| Not applicable | 0 (0) | 0 (0) | 0 (0) |
| **What is done if someone from the household of the index case is not home and they cannot be screened, n (%)** | | | |
| Visit the household later that day or on a subsequent day | 8 (22.9) | 23 (60.5) | 31 (42.5) |
| Schedule an appointment with the household members to return | 25 (71.4) | 19 (50.0) | 44 (60.3) |
| Do not return | 1 (2.9) | 3 (7.9) | 4 (5.5) |
| Other | 1 (2.9) | 0 (0) | 1 (1.4) |
| **Challenges in conducting screening in the community, n (%)** | | | |
| No challenge | 8 (25.8) | 3 (10.0) | 11 (18.0) |
| Language barrier | 1 (3.2) | 0 | 1 (1.6) |
| Insufficient information from patient/uncooperative patient | 3 (9.7) | 9 (30.0) | 12 (19.7) |
| Difficult or unable to contact/find patient | 7 (22.6) | 16 (53.3) | 23 (31.5) |
| Insufficient funding | 9 (29.0) | 1 (3.3) | 10 (16.4) |
| Insufficient manpower | 8 (25.8) | 0 (0) | 8 (13.1) |
| Weather and transportation difficulties | 3 (9.7) | 5 (16.7) | 8 (13.1) |

## **Supplementary Table 10: Foci response activities (Survey)**

| **Information on response activities** | **Health stakeholders/ staff** | **FHSPs** | **Total** |
| --- | --- | --- | --- |
| **Type of response activities triggered when a malaria case or focus is identified, n (%)** | | | |
| Raising awareness about causes of malaria transmission | 27 (79.4) | 29 (76.3) | 56 (77.8) |
| Raising awareness about malaria prevention | 30 (88.2) | 31 (81.6) | 61 (84.7) |
| Providing additional vector control if needed | 23 (67.7) | 10 (26.3) | 33 (45.8) |
| Entomological surveillance | 15 (44.1) | 4 (10.5) | 19 (26.4) |
| Spot checks for mosquito breeding grounds | 6 (17.7) | 4 (10.5) | 10 (13.9) |
| **Information gained from reactive surveillance influenced the kinds of response activities, n (%)** | | | |
| Yes | 34 (97.1) | 23 (67.7) | 57 (82.6) |
| No | 1 (2.9) | 11 (32.4) | 12 (17.4) |
| **Timing of commencement of response activities after a malaria case is initially reported, n (%)** | | | |
| Within 24 hours | 7 (20.0) | 18 (47.4) | 25 (34.3) |
| Within 48 hours | 6 (17.1) | 7 (18.4) | 13 (17.8) |
| Within 72 hours | 3 (8.6) | 5 (13.2) | 8 (11.0) |
| Within 7 days | 15 (42.9) | 6 (15.8) | 21 (28.8) |
| Within 14 days | 1 (2.9) | 1 (2.6) | 2 (2.7) |
| Within 28 days | 2 (5.7) | 1 (2.6) | 3 (4.1) |
| After 28 days | 1 (2.9) | 0 | 1 (1.4) |
| **Specific RASR targeted to MMP including forest-goers, n (%)** | | | |
| Yes | 29 (82.9) | -na- | 29 (82.9) |
| No | 6 (17.1) | -na- | 6 (17.1) |
| **Current RASR are sufficient for targeting *P. vivax* malaria, n (%)** | | | |
| Yes | 32 (91.4) | -na- | 32 (91.4) |
| No | 3 (8.6) | -na- | 3 (8.6) |
